# Local delivery of OSK factors enables partial cellular reprogramming to mitigate osteoarthritis and cartilage fibrosis

**DOI:** 10.1038/s12276-026-01662-x

**Published:** 2026-03-05

**Authors:** Yi-Wei Liu, Jing-Tao Zou, Jiang-Shan Gong, Ling Jin, Jia Cao, Ze-Hui He, Yu-Xuan Qian, Xin Wang, Mei-Dan Wan, Xin-Yue Hu, Chun-Gu Hong, Wei Du, Chun-Yuan Chen, Hong-Ji Liu, Hui Xie, Zhen-Xing Wang

**Affiliations:** 1 Department of Orthopedics, Movement System Injury and Repair Research Center, Xiangya Hospital, Central South University, Changsha, China; 2 Hunan Key Laboratory of Angmedicine, Changsha, China; 3 Department of Neurology, Xiangya Hospital, Central South University, Changsha, China; 4 National Clinical Research Center for Geriatric Disease, Xiangya Hospital, Changsha, China; 5 Department of Respiratory Medicine, Xiangya Hospital, Central South University, Changsha, China

**Keywords:** Experimental models of disease, Diseases

## Abstract

Osteoarthritis (OA) is a prevalent joint disease with a complex etiology, involving epigenetic alterations. Recent studies have suggested the potential of Oct4, Sox2 and Klf4 (OSK) in rejuvenating adult cells and facilitating tissue repair, but their specific role in OA pathophysiology and treatment remains unclear. Here we employed an adeno-associated virus (AAV) vector to achieve ectopic expression of OSK (AAV-OSK). Chondrocytes expressing OSK retained chondrocyte-specific markers with no increase in stemness-associated genes. AAV-OSK significantly preserved chondrocyte vitality in an inflammatory environment and counteracted the upregulation of osteogenic genes during OG differentiation. In OA murine models, AAV-OSK administration led to a notable improvement in cartilage integrity, a reduction in subchondral bone thickening and promoted the hyalinization of fibrocartilage. Furthermore, chondrocyte senescence and DNA methyltransferase expression were markedly diminished in the AAV-OSK group. Tet methylcytosine dioxygenase 2 was identified as a pivotal factor underlying the benefits of OSK-driven cartilage regeneration. Collectively, our study underscores that OSK expression within the knee joint modulates epigenetic alterations, mitigating OA progression and cartilage fibrosis through partial reprogramming, highlighting its therapeutic promise for comprehensive OA intervention.

## OSK expression reverses osteoarthritis through epigenetic modulation

Osteoarthritis (OA) is a common joint disease that causes pain and stiffness. Here researchers explored a new approach using epigenetics to treat OA. They focused on three factors, Oct4, Sox2 and Klf4 (OSK), which can reprogram cells to a more youthful state. The study used adeno-associated virus vectors to deliver OSK into mouse joint tissues. This method aimed to reduce inflammation and prevent cell death in cartilage cells (chondrocytes). The researchers found that OSK helped maintain healthy cartilage by reducing harmful changes and promoting repair. The results showed that OSK could slow down OA progression by altering DNA methylation. This suggests that OSK might rejuvenate cartilage cells and improve joint health. The study concludes that OSK has potential as a new treatment for OA, with future research needed to refine this approach for human use.

This summary was initially drafted using artificial intelligence, then revised and fact-checked by the author.

## Introduction

Osteoarthritis (OA) represents one of the most prevalent degenerative joint disorders, affecting millions worldwide with profound clinical and socioeconomic consequences^1^. It is characterized by the progressive deterioration of articular cartilage, abnormal subchondral bone remodeling, osteophyte formation, persistent inflammation and cartilage fibrosis^2,3^. The pathogenesis of OA is intricately linked with the aberrant differentiation and heightened catabolic activity of chondrocytes, typically driven by aging or excessive mechanical stress^4^. Given the avascular and aneural nature of the articular cartilage, its regenerative capability post-injury remains particularly limited^5^. When cartilage is damaged, chondrocytes abnormally proliferate and produce type I collagen (COL1), forming thick fibrocartilage-like tissue with poor mechanical properties, worsening OA symptoms^6,7^. Conventional OA treatments, including analgesics, physiotherapy and surgical interventions such as joint replacements, primarily provide symptomatic relief rather than addressing the underlying pathogenic mechanisms driving OA^8^. This underscores an urgent need for innovative therapeutic strategies that target the underlying pathogenic mechanisms of OA and enhance the intrinsic defensive mechanisms of joint tissues.

Epigenetics, defined as heritable alterations in gene expression without DNA sequence modifications, have been shown to modulate gene expression and maintain cellular identity^9^. Emerging studies have suggested that epigenetic dysregulation contributes to OA pathogenesis^10–13^. Key epigenetic players, including DNA methylation, histone modifications and noncoding RNAs, are implicated in modulating gene expression patterns and cellular phenotypes associated with OA^10,13,14^. For instance, DNA methylation intricately orchestrates cartilage homeostasis by governing the transcriptional dynamics of pivotal cartilage-related genes, such as *COL2A1* (cartilage anabolism) and *MMP13* (ECM degradation), subsequently influencing chondrocyte differentiation and OA progression^15^. Notably, a dysregulated balance in DNA methyltransferase (DNMT) levels, as evidenced by augmented DNMT1 and DNMT3a alongside diminished DNMT3b, has been observed in the cartilage of patients with OA and in the destabilization of the medial meniscus (DMM) mouse model, underscoring their pivotal roles in OA onset^16^. The relationship between OA and its associated epigenetic alterations suggests a promising avenue for the development of therapeutic strategies aimed at mitigating disease progression.

The concept of reprogramming, specifically through the induction of select transcriptional factors, has emerged as a compelling strategy for epigenetic intervention in OA^17^. This approach, which can be initiated by methods such as nuclear fusion, the introduction of transcription factors or small-molecule compounds, has the potential to reset epigenetic marks. It enables the rejuvenation of cells and the regeneration of tissues that have limited intrinsic repair capacity^18,19^. The famed quartet of *Oct4*, *Sox2*, *Klf4* and *c-Myc* (OSKM) has been explored for their ability to reverse cellular aging^20,21^. However, complete dedifferentiation, which entails the transformation of somatic cells into a pluripotent state, is associated with the tumorigenic risks^22^. Thus, partial reprogramming strategies using a subset of these factors, such as *Oct4*, *Sox2* and *Klf4* (OSK), have been proposed as a safer alternative^23^, demonstrating potential in reducing aging markers without reverting cells entirely to pluripotent states^23,24^. Recent evidence indicates that rejuvenation of nucleus pulposus cells can reverse intervertebral disc degeneration^25^. Nevertheless, therapeutic strategies focusing on partial reprogramming and maintaining the inherent traits of chondrocytes, a promising frontier for OA intervention^21^, have not yet been fully explored.

Nevertheless, a critical gap in current research is the feasibility and effectiveness of locally delivering partial reprogramming factors for epigenetic modulation within the joint microenvironment. This study aims to evaluate the effects of OSK-mediated reprogramming within joint tissues, assessing its therapeutic capacity for OA. We employed the adeno-associated virus (AAV) vectors for precise OSK delivery, utilizing both destabilization of the DMM and anterior cruciate ligament transection (ACLT) mouse models to discern both preventive and therapeutic avenues for OA. Furthermore, we hypothesized that overexpression of OSK factors can induce cellular reprogramming and facilitate the restoration of normal epigenetic patterns in chondrocytes. By unlocking the therapeutic potential of reprogramming in OA through local epigenetic modulation, this study could pave the way for the development of effective and safe epigenetic-based therapies for this prevalent joint disease.

## Materials and methods

### Construction and preparation of AAV-OSK

To generate the AAV expressing the OSK factors, a single plasmid was engineered to concurrently express Oct4, Sox2 and Klf4. These three factors were linked together using the self-cleaving P2A and T2A peptide sequences, ensuring their equimolar expression when transduced into target cells. Specifically, the sequence arrangement was Oct4-P2A-Sox2-T2A-Klf4 (Supplementary Fig. 1b). For the transcriptional control of the OSK genes, the EFS promoter, which is known for its robust expression, was employed. Once the OSK construct had been correctly assembled and its sequence verified, it was then packaged into the AAV2 serotype vectors. The AAV2 serotype was chosen owing to its efficient transduction properties in cartilage-associated cell types^26^.

To serve as a control for our experiments, an AAV2 vector expressing the enhanced green fluorescent protein (EGFP) was also constructed (Supplementary Fig. 1a). This approach permitted a visual and quantitative assessment of the transduction efficiency, thereby ensuring that any observed effects in our study could be attributed to the OSK expression and not to the viral vector itself. Once the constructs were prepared, they were transfected into HEK293T cell line for viral packaging. Subsequently, the cells were harvested, and the AAV2 viral particles were purified using a series of centrifugation and column chromatography steps. The final viral titer (>1 × 10^12^ genome copies per ml) was determined using qPCR, thereby ensuring a consistent and high concentration of viral particles for subsequent experiments. The AAV-OSK and AAV-Mock were manufactured by Haixing Biosciences (HX20220615JFY02A and HX20211115J01, respectively).

### Cell isolation and culture

Primary mouse chondrocytes were isolated by dissecting articular cartilage from 5–6-day-old mice and treated with 0.25% trypsin (12604021, Thermo Fisher) for 45 min at 37 °C, and then in 0.5 mg/ml collagenase II (17101015, Gibco) in Dulbecco’s modified Eagle’s medium (DMEM; 10567022, Gibco) overnight at 37 °C. The cell suspensions were filtered through a 70-μm filter and centrifuged, and then the cell pellets were resuspended and plated on Petri dishes^27^.

ATDC5 cells, a well-established chondrogenic cell line, were obtained from Riken BioResource Center (RCB0565, Tsukuba). The cells were routinely maintained in DMEM and Ham’s F-12 Nutrient Mix (DMEM:F-12; 12634010, Gibco), supplemented with 10% fetal bovine serum (FBS; A5669701, Gibco) and 1% penicillin/streptomycin (P1400, Solarbio). The culture environment was maintained at 37 °C in a humidified atmosphere containing 5% CO_2_.

### Cell models and treatments

To simulate the inflammatory infiltration observed in OA conditions, ATDC5 cells were exposed to 10 ng/ml interleukin-1β (IL-1β) recombinant protein (Ag23461, Proteintech). The selected concentration and exposure duration were determined through preliminary titration experiments to ensure an optimal inflammatory response without compromising cell viability.

To induce apoptosis in chondrocytes, an essential pathological feature in OA, the cells were treated with 10 ngml of tumor necrosis factor (TNF) recombinant protein (Ag11413, Proteintech) for 24 h.

Alongside the induction of inflammation and apoptosis, ATDC5 cells were concurrently infected with AAV-OSK or AAV-Mock. The multiplicity of infection was previously screened by gradient concentration tests.

To induce osteogenic differentiation in ATDC5 cells, a differentiation medium containing 50 µM α-ascorbate-2-phosphate, 10 mM β-glycerophosphate and 0.1 µM dexamethasone was prepared. The cells were exposed to the differentiation cocktail (MUXMX-90021, Cyagen Biosciences) following the manufacturer’s instructions, with medium changes every 48 h.

The slides with cells were fixed in 4% paraformaldehyde (PFA; BL539A, Biosharp) for 15 min. Subsequent processing involved immunocytochemistry (ICC) staining.

### RNA isolation and cDNA synthesis

Following a 48-h treatment period, the cells were collected and total RNA was extracted from the resultant cell pellets employing TRIzol reagent (AG21102, Accurate Biology) according to the manufacturer’s protocol. To prevent DNA contamination, the RNA samples were treated with DNase I (Z3585, Promega). The concentration and purity of the isolated RNA were assessed using a microplate reader (Varioskan LUX, Thermo Scientific), and only samples with an A260/A280 ratio within the range of 1.8–2.0 were considered suitable for downstream applications. Subsequently, 1 μg of purified RNA was subjected to reverse transcription using a reverse transcription kit (E047, Novoprotein).

### RT–qPCR amplification and analysis

QPCR amplifications were conducted on a Promega FTC-3000 instrument (Funglyn Biotech Inc.) employing SYBR Green master mix (B21202, Selleck). The primers for the target genes were custom designed and synthesized by Sangon Biotech. The primer sequences utilized for qPCR analyses are provided in Supplementary Table 1.

Postamplification, a melt curve analysis was routinely performed to confirm the specificity of the PCR products. The threshold cycle (*Ct*) value for each tested gene was extracted and normalized against the *Ct* value of the housekeeping gene, glyceraldehyde-3-phosphate dehydrogenase (*Gapdh*), using the ΔΔ*Ct* method. The relative fold changes in gene expression were determined using the 2^−ΔΔ*Ct*^ calculation.

All RT–qPCR experiments were performed in triplicate to ensure consistency and reproducibility. Variations between experimental samples were statistically analyzed and presented accordingly.

### Flow cytometry

Flow cytometry was employed to detect cell apoptosis in ATDC5 cells treated with TNF. After treatment, the cells were collected by trypsinization (T1300, Solarbio) and stained with Fluor647 Annexin V and propidium iodide (PI) according to the manufacturer’s instructions (Annexin V-Alexa Fluor647/PI Apoptosis Detection kit; 40304ES20, Yeasen). Then the cells were analyzed using a Becton Dickinson FACSCantoTM Ⅱ within 1 h.

### Generation of Col2α1-CreERT;Rosa26-tdTomato mice

*Col2α1-CreERT* mice (006774, Jackson Laboratory) were crossbred with the *Rosa26-tdTomato* strain (007909, Jackson Laboratory) to generate *Col2α1-CreERT;Rosa26-tdTomato* reporter mice. Two-month-old mice were treated with 75 mg/kg tamoxifen (T5648, Sigma) for 5 days, then underwent surgery and intervention. Before their utilization, all transgenic mice were subjected to rigorous genotyping procedures (Supplementary Fig. 2).

### Surgical OA mice models and treatments

Twelve-week-old male C57/BL/6 wild-type mice were selected for the induction of OA via either DMM or ACLT surgical procedure. For comparative purposes, a cohort of mice underwent a sham operation. To minimize the number of animals used in accordance with the 3Rs principle, some of the mice in the sham group also served as controls for baseline comparisons across different experimental conditions. The DMM and ACLT surgical procedures were conducted in accordance with established protocols to ensure consistency and replicability^28,29^.

Subsequently, >1 × 10^11^ genome copies of AAV were meticulously injected into the articular cavity of each mouse to ascertain its therapeutic potential. For both DMM/ACLT and sham-operated groups, the sample size was maintained at 6 - 8 mice per group to ensure statistically significant findings. The articular cartilage from DMM-induced OA mice treated with AAV-MOCK or AAV-OSK were conducted whole-genome bisulfite sequencing (WGBS) to analyze methylation patterns. All breeding, housing and experimental interventions involving these animals were conducted in accordance with protocols approved by the Ethical Committee at Xiangya Hospital, Central South University (approval number 202101006). In addition, all methods were conducted in line with the Guidelines for the Care and Use of Laboratory Animals endorsed by the same committee. In all stages of the procedure, meticulous care was taken to minimize any undue distress or discomfort to the animals, in accordance with the principles of the 3Rs: Reduction, Replacement and Refinement.

### Intra-articular injection technique

Intra-articular injections into the knee joint were performed using the patellar tendon approach. To ensure accurate needle placement, the patellar tendon was visually identified through a small longitudinal skin incision of approximately 5 mm. The injection volume was limited to 20 μl per joint and injections were completed without evidence of joint leakage.

### siRNA preparation

siRNA was purchased from Ribobio (Guangzhou, China) for *Tet2*. Intra-articular delivery of siRNA was performed at a concentration of 1.5 nmol/20 μl, as previously described^30^.

### Mechanical nociception hyperalgesia test (Von Frey test)

Mice were adapted by placing them in individual cages for a 30-min acclimation period. The sensitivity of the hind paw to punctate static mechanical stimulation was assessed using calibrated von Frey nylon filaments (Ugo Basile). The filaments were systematically applied to the midplantar surface of the hind paw through the mesh cage flooring. The force threshold required to elicit a 50% positive withdrawal response was documented as the paw withdrawal threshold, which signifies mechanical sensitivity.

### Balance beam test

The balance beam test was used to evaluate the proprioceptive balance and agility of mice^31^. Mice were introduced at one end of a wooden strip (0.9 cm × 0.9 cm × 50 cm) situated 40 cm above the ground. To encourage the mice to traverse the beam, a darkened target box was placed at the opposite end, capitalizing on their natural inclination toward dark and secure environments. The total time required for the mice to traverse the beam was recorded. The duration was then used to calculate the crossing speed. Each mouse was subjected to three trials daily, with a 20-min interval between each trial. This regimen was sustained for five consecutive days.

### Grip strength test

To assess the muscular strength and endurance of the mice, a grip strength test was employed^31^. In brief, the mice were prompted to grasp a suspended wire (9.0 mm in diameter, 50.0 mm in length), elevated 40.0 cm from the ground. The time span before the mice lost their grip, termed as the grip latency, was recorded. To ensure the safety of the mice, a maximum observation limit of 5 min was set. Each mouse underwent three trials per day, with a 20-min rest interval between successive trials, for a total of five consecutive days.

### Micro-CT analysis of knee joints

The knee joints were excised and immediately immersed in 4% PFA, where they were fixed for 48 h at ambient conditions to ensure optimal preservation. Subsequently, the fixed knee joints underwent high-resolution microcomputed tomography (micro-CT) scanning using the vivaCT 80 system (SCANCO Medical AG). The scanning parameters were set to a voltage of 70 kV and a current of 400 µA, with an image resolution of 11.4 µm per pixel.

The acquired two-dimensional scans were reconstructed employing Skyscan NRecon software, delivering detailed cross-sectional views of the knee architecture. The CTAn software suite was then used for quantitative image analysis. To provide a comprehensive visual appreciation, three-dimensional (3D) images of the joints and the tibial subchondral bone were generated using the CTVol software.

The sagittal images, which were specifically targeted at the tibial subchondral bone, served as the focal point for the 3D histomorphometric analyses. The key structural parameter assessed was the bone volume fraction (bone volume/tissue volume, BV/TV). To ensure a comprehensive representation of the medial subchondral compartment, the analysis encompassed 20 sequential images of the medial tibial plateau.

### Histopathological analysis

At 8 weeks postoperatively, the knees from both the surgery- and sham-operated groups were collected. The collected samples underwent a comprehensive histological analysis, with the primary objective being the assessment of OA changes. Histological grading was scrupulously performed employing the Osteoarthritis Research Society International (OARSI) histopathological grading system (Supplementary Table 2). This system provides a detailed stratification of the progression of OA based on observable morphological changes in the articular cartilage^32^.

### Histological analysis of joints

The knee joints were fixed in 4% PFA for 48 h and then decalcified in 0.5 M EDTA for 7 days. The joint samples were dehydrated via a graded ethanol, cleared in xylene and embedded in paraffin. The paraffin-embedded samples were sectioned at a thickness of 5 μm and subsequently subjected to hematoxylin and eosin (H&E) staining using reagents procured from Servicebio (G1076). For immunohistochemistry staining, antibodies against COL2α1 (PA1-26206, Invitrogen), ACAN (GB11373, Servicebio), COLX (ab182563, Abcam), MMP13 (PA5-27242, Invitrogen), P21 (GB11153, Servicebio), DNMT3a (ab188470, Abcam) and the corresponding horseradish peroxidase (HRP)-conjugated secondary antibody (GB23303, Servicebio) were utilized.

### Immunofluorescence staining

The knee joints were fixed in 4% PFA for 48 h and then decalcified in 0.5 M EDTA for 7 days. For the preparation of frozen sections, samples underwent dehydration in 30% sucrose and subsequently embedded in an optimal cutting temperature (OCT) compound. Primary antibodies against SOX2 (14-9811-82, Thermo Fisher), IL-1 (GB11113, Servicebio), RUNX2 (12556, CST), OSX (sc393325, Santa Cruz) and OCN (GB11233, Servicebio) were utilized. Subsequently, secondary antibodies were employed, specifically Alexa Fluor 488-conjugated AffiniPure goat anti-Rabbit IgG (111-545-144, Jackson ImmunoResearch), Alexa Fluor 594-conjugated AffiniPure donkey anti-Rat IgG (712-585-153, Jackson ImmunoResearch) and Alexa Fluor647-conjugated AffiniPure goat anti-Rabbit IgG (111-605-008, Jackson ImmunoResearch).

### SO-FG staining for cartilage analysis

Safranin O-Fast Green (SO-FG) staining was performed to examine proteoglycans in cartilage. The tissue sections were initially stained with fast green (G1053, Servicebio) for 5 min. Subsequently, a 10 s rinse was conducted with 1% hydrochloric acid in 70% ethanol solution (v/v) used for differentiation to remove excess stain and enhance contrast. Subsequently, the slices were incubated in SO solution for 3 min. Poststaining, the tissues were dehydrated using ethanol and mounted. Visual inspection and image acquisition were performed using a BX53 microscope (Olympus).

### WGBS

Genomic DNA (gDNA) was extracted from articular cartilage and utilized for WGBS library preparation using the AceGen Bisulfite-Seq Library Prep Kit (AceGen, AG0311) adhering to the manufacturer’s protocol. Following bisulfite treatment, the DNA was amplified in 10 cycles of PCR with Illumina 8 bp dual index primers. The constructed WGBS libraries were then analyzed with an Agilent 2100 Bioanalyzer and finally sequenced on Illumina platforms using a 150× two paired-end sequencing protocol. The FastQC tool was used to perform basic statistical analysis on the quality of the raw reads. BSMAP software was used to map the bisulfite sequences to the reference genome. Differentially methylated regions (DMRs) were identified using metilene (v0.2-7). Based on the results of DMR annotation and Kyoto Encyclopedia of Genes and Genomes (KEGG)^33^ annotation, functional enrichment analysis was performed on genes whose gene bodies or 2 kb upstream or downstream regions overlapped with a DMR.

### Construction and evaluation of the DNA methylation age model

Genome-wide DNA methylation profiles of 255 mouse samples, encompassing approximately 1.9 million CpG sites, were obtained from the GEO dataset GSE80672^34^. From these data, methylation levels and coverage depths at the 90 CpG sites reported in the reference study were extracted. Sites with coverage depths >4× were retained and missing values were imputed using the *K*-nearest neighbor algorithm. The resulting dataset was randomly partitioned into training and validation subsets at an 8:2 ratio to construct and evaluate the methylation age prediction model using an elastic net regression framework.

Following the established protocol^34^, predicted methylation ages were fitted against chronological ages to calibrate the model. For this study, methylation data from six experimental samples were processed using identical filtering criteria (coverage >4×), and missing values were imputed using *K*-nearest neighbor. The methylation age of each sample was subsequently estimated using the trained elastic net model.

### Statistical analysis

Data were expressed as means ± s.d. To assess the statistical significance between groups, either a Student’s *t*-test or analysis of variance (ANOVA) was employed, followed by a Bonferroni post hoc test to correct for multiple comparisons. For nonparametric data, the Kruskal–Wallis test was used. All statistical evaluations were conducted using GraphPad Prism version 8.0 software. A probability value (*P* value) of less than 0.05 was considered statistically significant. Significance levels were denoted as *P ≤ 0.05, **P ≤ 0.01, ***P ≤ 0.001 and ****P < 0.0001.

## Results

### OSK exhibits anti-inflammatory and anti-apoptotic effects in chondrocytes

**Fig. 1:**
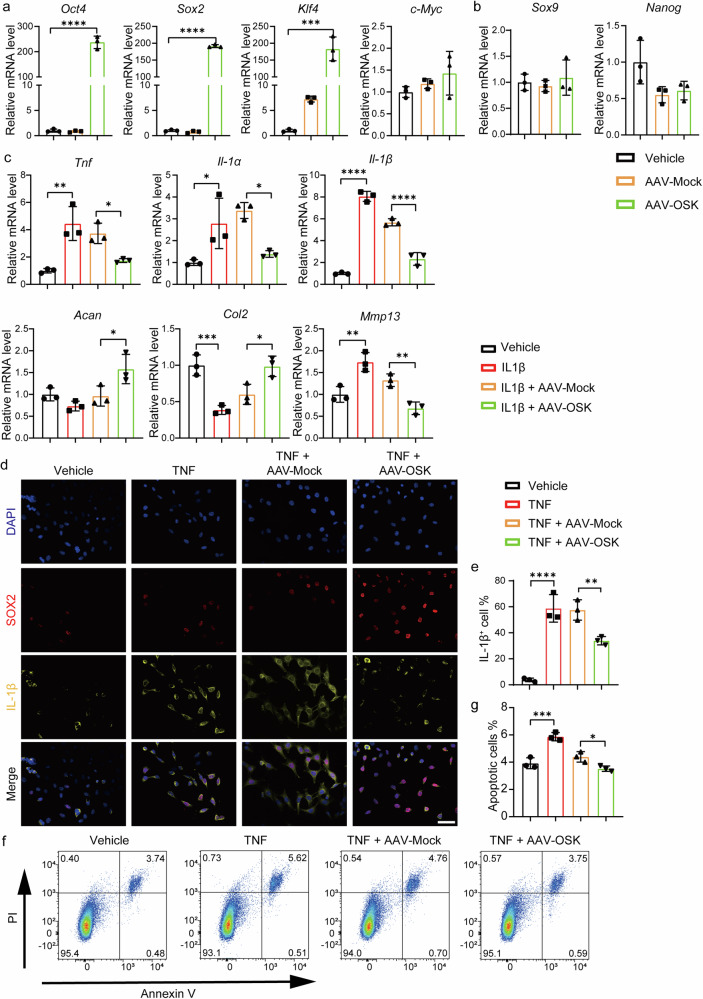
OSK exhibits anti-inflammatory and anti-apoptotic effects in chondrocytes. **a**, **b** RT–qPCR analysis of *Oct4*, *Sox2*, *klf4* and *c-Myc* (**a**) and *Sox9*, *Nanog* (**b**) in primary chondrocytes receiving either AAV-Mock or AAV-OSK treatments. *n* = 3 per group. **c** RT–qPCR analysis of inflammatory genes (*Tnf*, *Il-1α* and *Il-1β*) and metabolic genes (*Acan*, *Col2* and *Mmp13*) in ATDC5 cell line under inflammatory environment induced by IL-1β for 24 h, after treatment with vehicle, AAV-Mock or AAV-OSK for 3 days. *n* = 3 per group. **d**, **e** ICC staining of SOX2 and IL-1β in ATDC5 cells under inflammatory conditions and different treatments (**d**) and the percentage of IL-1β positive cells (**e**). *n* = 3 per group. Scale bar, 50 μm. **f** Flow cytometry assay of apoptosis in ATDC5 cells induced by TNF, treated with vehicle, AAV-Mock or AAV-OSK. **g** Quantification of the ratios of apoptotic (Annexin V^+^/PI^+^) cells in **f**. *n* = 3 per group. Data are presented as mean ± s.d. and analyzed using one-way ANOVA with Bonferroni post hoc test. *P < 0.05, **P < 0.01, ***P < 0.001, ****P < 0.0001.

To explore the potential of OSK (*Oct4*, *Sox2* and *Klf4*) in cellular reprogramming for OA therapy, a robust adeno-associated virus 2 (AAV2) system was employed to co-express these factors in chondrocytes (Supplementary Fig. 1). A crucial step in validating the therapeutic applicability of OSK is to confirm that these factors can be incorporated into chondrocytes without disrupting their inherent cellular identity. In our experiments, primary mouse chondrocytes were transduced with either an AAV2 vector encoding OSK (AAV-OSK) or a control vector expressing green fluorescent protein (GFP; AAV-Mock). Following transduction, there was a notable increase in the transcription levels of *Oct4*, *Sox2* and *Klf4*, whereas the levels of *c-Myc* remained stable in comparison to those in AAV-Mock transduced controls (Fig. 1a). In addition, despite the pronounced induction of OSK, the characteristic chondrogenic phenotype was persisted, as evidenced by the absence of the chondrocytic marker SRY-box transcription factor 9 (*Sox9*)^35^ and pluripotency marker NANOG homeobox (*Nanog*)^36^ post-transfection (Fig. 1b). These findings confirm that OSK transduction preserves the chondrocytic integrity, maintaining their cellular identity despite the expression of reprogramming factors.

OA progression is closely linked to biomechanical strain and subsequent inflammation^37,38^. This strain-triggered inflammation often presents via the secretion of proinflammatory cytokines, such as IL-1β, which subsequently initiates cartilage matrix degradation^39^. To investigate the impact of OSK on this inflammatory cascade, primary mouse chondrocytes were treated with recombinant IL-1β alongside either AAV-OSK or AAV-Mock. RT–qPCR revealed that IL-1β predictably triggered an elevation in transcripts of proinflammatory cytokines, including *Tnf*, *Il-1α* and *Il-1β* (Fig. 1c). This inflammation was concomitant with the suppression of cartilage anabolic factors, aggrecan (*Acan*) and collagen type II (*Col2*) and the elevation of catabolic marker, collagen type I (*Col1*) and matrix metallopeptidase 13 (*Mmp13*) (Fig. 1c), whereas chemokines *Cxcl1* and *Ccl2* remained unchanged (Supplementary Fig. 3). Notably, OSK overexpression significantly attenuated the IL-1β induced proinflammatory response, while restoring the expression of anabolic markers and reducing the levels of degradation markers (Fig. 1c).

To further validate the anti-inflammatory effect of OSK on chondrocytes, a mouse chondrogenic cell line ATDC5 was treated with recombinant TNF and transduced with either AAV-OSK or AAV-Mock. ICC staining confirmed the increased SOX2 expression in the AAV-OSK-treated ATDC5 cells, indicating successful OSK transduction (Fig. 1d). In addition, ICC staining for IL-1β showed a marked reduction in this proinflammatory factor following OSK transduction (Fig. 1d,e). Furthermore, we also analyzed the anti-apoptotic effect of OSK in ATDC5 cells. Flow cytometry analysis revealed a notable reduction in the percentage of apoptotic cells (Annexin V^+^/PI^+^) after OSK transduction compared to controls (Fig. 1f,g). These results delineate the anti-inflammatory and anti-apoptotic effects of OSK in chondrocytes, highlighting its potential as an effective therapeutic for OA.

### OSK counteracts osteogenic transition and preserves chondrocyte phenotype

**Fig. 2:**
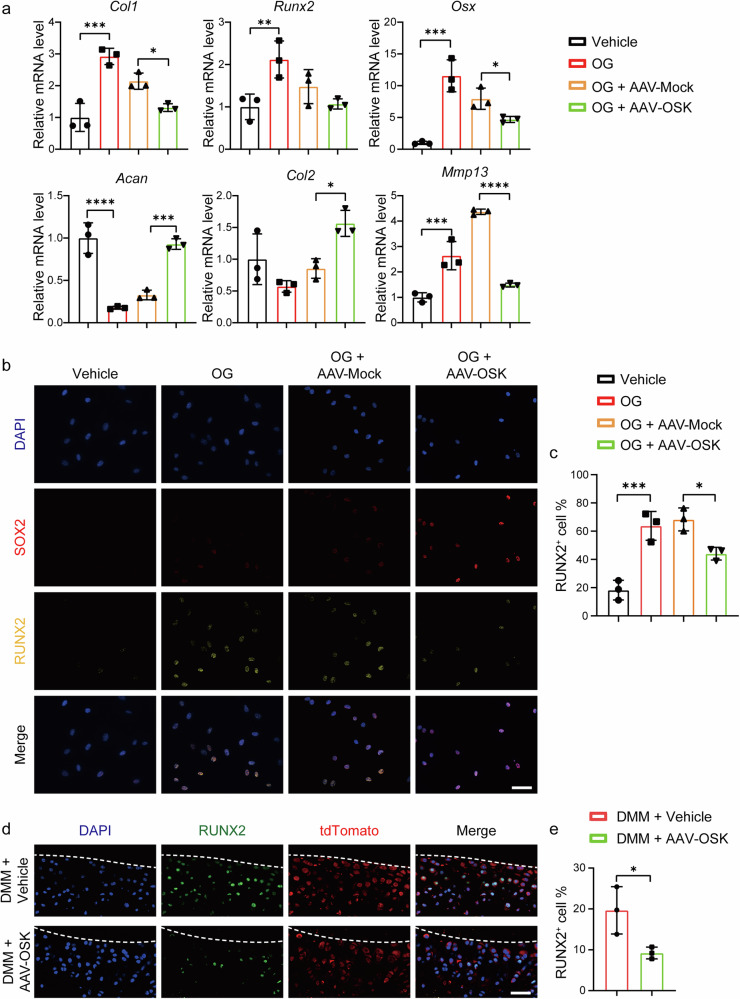
OSK counteract osteogenic transition and preserve chondrocyte phenotype. **a** RT–qPCR analysis of osteogenic genes (*Col1*, *Runx2* and *Osx*) and metabolic genes (*Acan*, *Col2* and *Mmp13*) in ATDC5 cells under osteogenic conditions for 3 days, after treatment with vehicle, AAV-Mock or AAV-OSK for 3 days. *n* = 3 per group. **b**, **c** ICC staining of SOX2 and RUNX2 in ATDC5 cells under osteogenic conditions (**b**) and the percentage of RUNX2-positive cells (**c**). *n* = 3 per group. Scale bar, 50 μm. **d** Immunofluorescence staining of RUNX2 in the joint of *Col2α1-CreERT;Rosa26-tdTomato* reporter mice following DMM surgery, treated with vehicle or AAV-OSK. Scale bar, 50 μm. **e** The percentage of RUNX2-positive cells in **d**. *n* = 3 per group. Data are presented as mean ± s.d. and analyzed using one-way ANOVA with Bonferroni post hoc test (**a** and **c**) or unpaired *t*-tests (**e**). *P < 0.05, **P < 0.01, ***P < 0.001, ****P < 0.0001.

OA is often associated with abnormal remodeling of subchondral bone and osteophyte development, leading to the deterioration of the tissue environment and chondrocyte dedifferentiation, which subsequently results in cartilage degradation and hyaline cartilage fibrosis^40,41^. To investigate whether OSK could maintain chondrogenic characteristics and prevent dedifferentiation of chondrocytes in an osteogenic environment, we employed an in vitro osteogenic environment that mimics the tissue conditions observed in OA^42^. Upon inducing osteogenesis (OG), ATDC5 cells underwent osteogenic transition, as evidenced by the upregulation of osteogenic-related genes, including *Col1*, runt-related transcription factor 2 (*Runx2*) and osterix (*Osx*), indicative of the dedifferentiation process (Fig. 2a). By contrast, when OSK was expressed, the transcript levels of these osteogenic markers were notably inhibited (Fig. 2a). In addition, OSK expression facilitated the maintenance of the cartilage synthesis factors (*Acan* and *Col2*) and led to a decline in the expression of the catabolic factors (*Mmp13*) in ATDC5 cells (Fig. 2a). Further ICC staining confirmed that under osteogenic conditions, OSK effectively inhibited the upregulation of RUNX2 and OSX (Fig. 2b,c and Supplementary Fig. 4a,b). This observation highlights the capacity of OSK to modulate key transcription factors involved in chondrocyte differentiation and osteogenic transition.

To assess the in vivo relevance of these findings, we utilized *Col2α1-CreERT;Rosa26-tdTomato* double transgenic mice (Supplementary Fig. 2), which allow for specific labeling of chondrocytes and their progenies with tdTomato fluorescence. Following the induction of OA via DMM surgery, we injected either vehicle or AAV-OSK into the articular cavities of these reporter mice. At 2 weeks postintervention, we observed a marked increase in the osteogenic and hypertrophic marker RUNX2^43^ within tdTomato-expressing chondrocytes of OA-afflicted mice (Fig. 2d, e). However, upon OSK administration, there was a discernible decrease in RUNX2 expression in tdTomato-positive chondrocytes (Fig. 2d, e), indicating a restoration of the chondrocyte phenotype.

Collectively, our findings highlight the pivotal role of OSK in mitigating osteogenic transition of chondrocytes and maintaining the chondrocyte phenotype under OA conditions.

### OSK expresses in chondrocytes without hyperproliferation

**Fig. 3:**
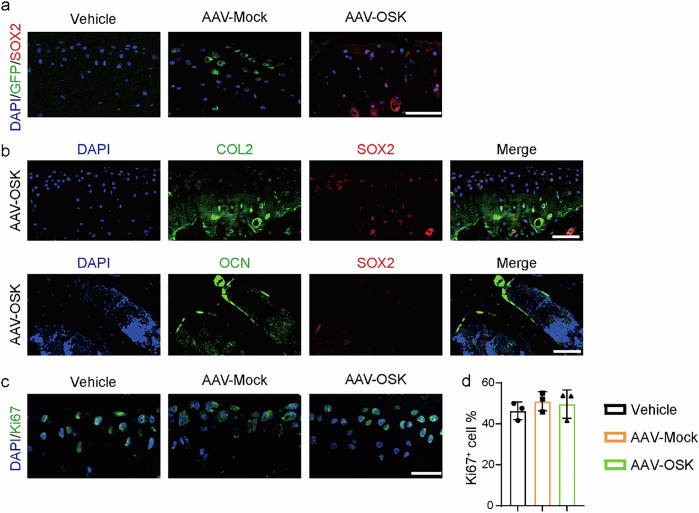
OSK expresses in chondrocytes without hyperproliferation. **a** Immunofluorescence staining of GFP or SOX2 in articular cartilage from mice treated with AAV-Mock or AAV-OSK. Scale bar, 50 μm. **b** Immunofluorescence staining of COL2 (upper), OCN (lower) and SOX2 in articular cartilage from mice treated with AAV-OSK. Scale bar, 50 μm. **c** Immunofluorescence staining of Ki67 in articular cartilage from mice treated with vehicle, AAV-Mock or AAV-OSK. Scale bar, 25 μm. **d** The percentage of Ki67-positive cells in **c**. *n* = 3 per group. Data are presented as mean ± s.d. and analyzed using one-way ANOVA with Bonferroni post hoc test. *P < 0.05.

Understanding the role of OSK in cartilage repair is essential, particularly in OA, a degenerative joint disease characterized by impaired regenerative capacity and abnormal chondrocyte activity^7^. To examine the potential benefits of OSK on chondrocytes, we injected AAV-OSK or AAV-Mock (integrated with GFP) into the articular cavities of wild-type mice. At 2 weeks postinjection, immunofluorescence analysis revealed a marked expression of GFP expression in the AAV-Mock-treated mice, whereas SOX2 was robustly expressed in the AAV-OSK-treated counterparts, indicating stable integration of OSK within the cartilaginous tissue (Fig. 3a).

Furthermore, to identify the specific cellular targets of AAV-OSK, immunofluorescence staining was performed following intra-articular injection. The analysis revealed robust co-localization of SOX2 with the chondrocyte-specific marker COL2 (Fig. 3b, upper), whereas SOX2 signal was minimally in OCN-positive subchondral bone (Fig. 3b, lower) and only minimal in the synovial region (Supplementary Fig. 5a). These findings indicate that OSK-mediated transgene expression primarily targets chondrocytes within the articular cartilage.

To gain insights into the proliferative status of chondrocytes post-OSK treatment, we employed Ki67 staining, a widely recognized marker for cellular proliferation^42^. As shown in Fig. 3c,d, the percentage of Ki67-positive chondrocytes did not differ between the AAV-Mock- and AAV-OSK-treated groups. These results imply that OSK does not induce excessive cellular proliferation, underscoring its potential therapeutic viability by facilitating tissue repair while mitigating the risk of hyperplasia.

### Amelioration of DMM- and ACLT-induced OA via OSK expression

**Fig. 4:**
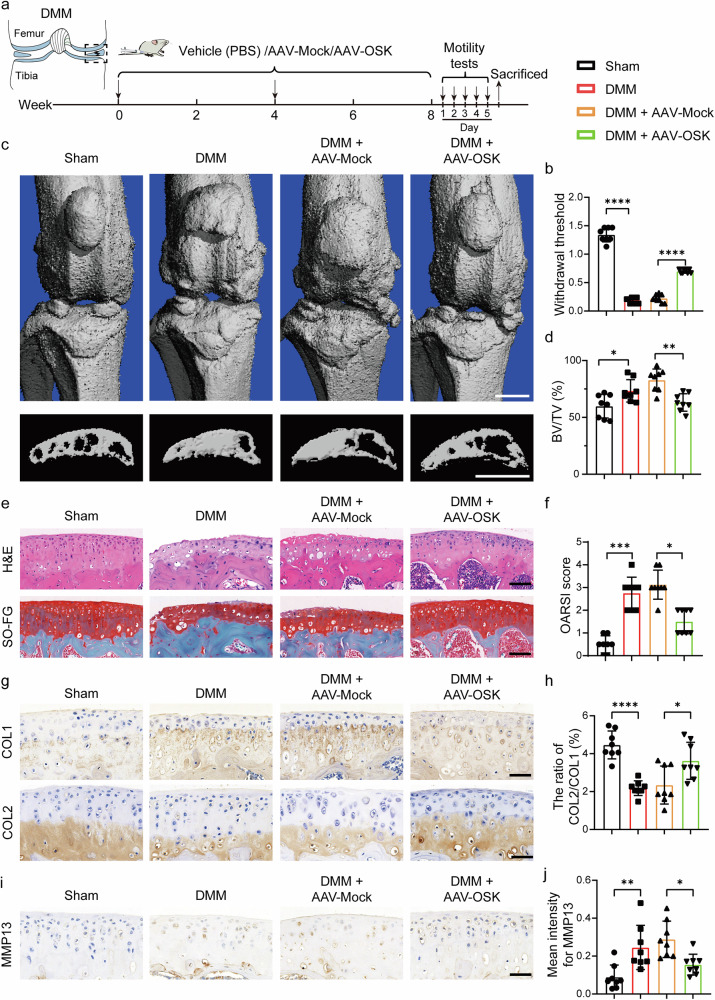
Amelioration of DMM-induced OA via OSK expression. **a** Diagram illustrating the timeline for DMM mouse modeling and intervention with OSK. **b** Pain measurement using the von Frey assay test. *n* = 8 per group. **c** Representative 3D reconstructed micro-CT images of the joint and sagittal views of tibia subchondral bone in treated mice 8 weeks after DMM surgery. Scale bar, 1 mm. **d** Quantitative analysis of the bone volume fraction of subchondral bone (BV/TV). *n* = 8 per group. **e** Representative images of H&E and SO-FG staining. Scale bar, 100 μm. **f** OARSI scores based on histological analysis of SO-FG staining. *n* = 8 per group. **g**, **h** Representative immunohistochemical staining of COL1 and COL2 (**g**) and quantification of the ratio of the positive area of COL2 to COL1 (**h**). Scale bar, 50 μm. *n* = 8 per group. **i**, **j** Representative immunohistochemical staining of MMP13 (**i**) and respective quantitative analysis of staining intensity (**j**). Scale bar, 50 μm. *n* = 8 per group. Data are presented as mean ± s.d. and analyzed using one-way ANOVA with Bonferroni post hoc test or nonparametric Kruskal–Wallis test (**f**). *P < 0.05, **P < 0.01, ***P < 0.001, ****P < 0.0001.

Given the above findings that OSK could benefit OA by counteracting inflammation and apoptosis while maintaining the chondrocytic phenotype, we sought to validate the protective role of OSK in surgical models of OA. Two prevalent surgical models, DMM and ACLT, were used to induce OA-like conditions^29^. Following model establishment, mice were treated with either vehicle, AAV-Mock or AAV-OSK into the joint cavity on the day of surgery and again at 4 weeks later. The effects of these interventions were analyzed after an 8-week period (Fig. 4a and Supplementary Fig. 7a).

Since functional outcomes often provide the earliest observable symptomatology in OA, we conducted the grip strength test and balance beam test on the mice weekly post-treatment. The results consistently showed that the AAV-OSK-treated cohorts exhibited improved performance compared to AAV-Mock-treated OA mice, suggesting attenuated OA severity or enhanced compensatory mechanisms in the presence of OSK (Supplementary Figs. 6a and 8a). Furthermore, to evaluate nociceptive responses, a mechanical nociception hyperalgesia test was conducted after 8-week treatment period. We found that OA mice (vehicle and AAV-Mock groups) exhibited lower paw withdrawal thresholds during the von Frey assay, indicative of heightened pain sensitivity (Fig. 4b and Supplementary Fig. 7b). Conversely, AAV-OSK intervention ameliorated this heightened nociceptive response, highlighting its potential analgesic properties (Fig. 4b and Supplementary Fig. 7b).

To gain insight into the microstructural changes, micro-CT was employed to visualize joint injuries. OA induction led to significant joint surface destruction and an increased volume fraction of subchondral bone (Fig. 4c,d and Supplementary Fig. 7c, d). However, OSK expression reversed these detriments, underscoring its reparative or protective capacities in the OA setting (Fig. 4c,d and Supplementary Fig. 7c, d).

To further investigate the potential protective effects of OSK on the structural integrity of cartilage, we performed histological evaluations using H&E and SO-FG staining. H&E staining revealed well-preserved chondrocyte morphology and an intact cartilage matrix in the OSK-treated group compared to the AAV-Mock-treated DMM and ACLT model groups (Fig. 4e and Supplementary Fig. 7e, upper). Furthermore, the SO-FG staining illustrated pronounced red staining, indicative of abundant proteoglycan content in the OSK-treated cartilage, which starkly contrasted with the reduced staining intensity observed in the AAV-Mock-treated counterparts (Fig. 4e and Supplementary Fig. 7e, lower). To quantify cartilage degradation, we used the OARSI scoring system, a reliable metric for assessing the severity of cartilage damage in OA. The OARSI scores for the OSK-treated group were notably lower than those of the control group (Fig. 4f and Supplementary Fig. 7f), indicating a lesser degree of cartilage degeneration. Moreover, the ratio of COL2 to COL1 was markedly decreased in the OA models but was significantly restored following OSK treatment (Fig. 4g,h and Supplementary Fig. 7g, h). In addition, immunohistochemical assays revealed that the enhanced expression of anabolic chondrogenic marker ACAN in OSK-treated joints (Supplementary Figs. 4b,c, 6b,c and 8b, c), along with a suppression of the catabolic protein MMP13 (Fig. 4i, j and Supplementary Fig. 7i, j) and the hypertrophic marker COLX (Supplementary Figs. 6d,e and 8d, e). These results indicate that OSK could restore the homeostatic balance within the OA environments.

In summary, our results illuminate the multiple therapeutic potentials of OSK in OA. It not only physiologically improves function and attenuates pain, but also induces molecular and structural changes that counteract the pathological progression of OA.

### OSK attenuates OA-driven epigenetic aging via DNA methylation modulation

**Fig. 5:**
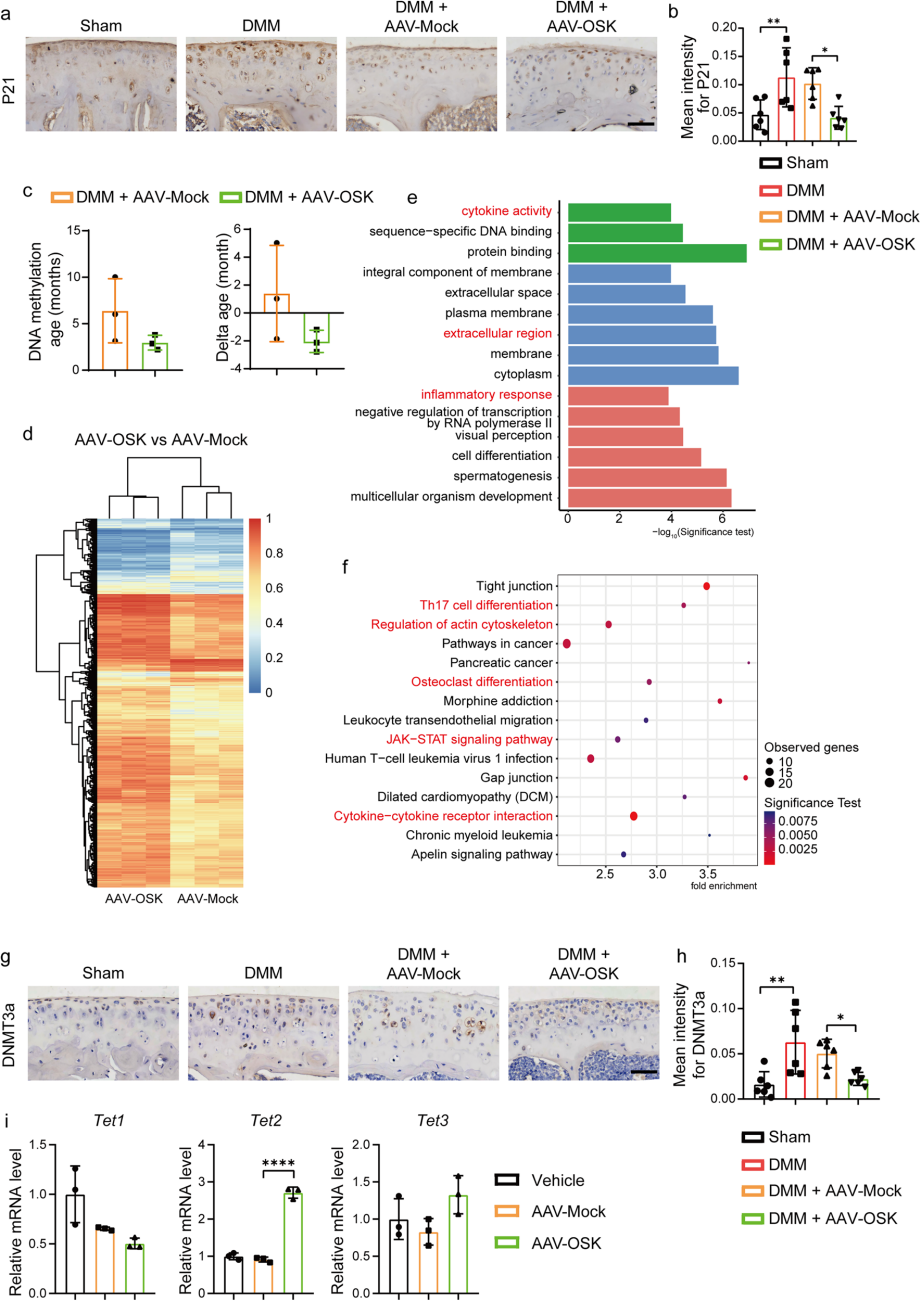
OSK attenuate OA-driven epigenetic aging via DNA methylation modulation. **a**, **b** Representative immunohistochemical staining of P21 (**a**) and quantitative analysis of staining intensity (**b**). Scale bar, 50 μm. *n* = 6 per group. **c** The discrepancy between predicted DNA methylation age and actual chronological age (delta age) of AAV-Mock and AAV-OSK-treated DMM mice. *n* = 3 per group. **d** A hierarchical clustered heat map of methylation levels of AAV-MOCK and AAV-OSK groups. **e**, **f** The top enriched biological process terms (**e**) and enriched KEGG pathways (**f**) showing altered promoter regions between AAV-Mock and AAV-OSK-treated DMM mice. **g**, **h** Representative immunohistochemical staining of DNMT3a (**g**) and quantitative analysis of staining intensity (**h**). Scale bar, 50 μm. *n* = 6 per group. **i** RT–qPCR analysis of *Tet1–3* expression in primary chondrocytes treated with vehicle, AAV-Mock or AAV-OSK. *n* = 3 per group. Data are presented as mean ± s.d. and analyzed using one-way ANOVA with Bonferroni post hoc test. *P < 0.05, **P < 0.01, ****P < 0.0001.

OA pathogenesis is intricately linked with the aging processes of articular cartilage^44^. A hallmark of this senescence in articular cells is the upregulation of cyclin-dependent kinase (CDK) inhibitors, such as P21^4^, which serve as cellular checkpoints to restrict aberrant proliferation and indicate cellular aging. Indeed, in the DMM model, we observed an elevated expression of P21 (Fig. 5a, b), suggesting increased chondrocyte senescence. However, intervention with OSK attenuated this upregulation (Fig. 5a, b), indicating its potential anti-aging effects on articular cells.

One of the pivotal mechanisms driving tissue aging is epigenetic modulation, particularly DNA methylation. Aberrant DNA methylation patterns have been implicated in the aging of various tissues, with heightened levels often indicative of cellular senescence^45^.DNA methylation, a stable epigenetic modification, contributes to aging and disease progression at the molecular level. In this study, WGBS was performed on articular cartilage from DMM-induced OA mice treated with AAV-MOCK or AAV-OSK to analyze methylation patterns. The epigenetic aging clock, or DNA methylation age, is a highly accurate biomarker of chronological age and a robust predictor of health outcomes, including mortality and cardiovascular disease^34,46^. Using the methylation age prediction method described in the article^34^, we found that the methylation age of joint cartilage in the AAV-OSK intervention group was reduced compared to that in the AAV-MOCK group. Moreover, the predicted methylation age of the AAV-OSK group was younger than its chronological age (delta age), indicating that OSK expression can rejuvenate chondrocytes and potentially reverse OA progression (Fig. 5c). To comprehensively investigate genome-wide DNA methylation alterations, we identified DMRs across all samples. Hierarchical clustering of these DMRs revealed distinct methylation profiles between the AAV-MOCK and AAV-OSK groups (Fig. 5d), indicating that OSK intervention markedly reshaped the global DNA methylation landscape. Gene ontology (GO) analysis revealed that the differentially methylated genes in promoter regions were enriched in functions of cytokine activity, extracellular region and inflammatory response, which were among the enriched terms altered in OA and modified by OSK treatment (Fig. 5e). KEGG pathway analysis further demonstrated that the differentially methylated genes were enriched in pathways such as Th17 cell differentiation, regulation of actin cytoskeleton, osteoclast differentiation, the JAK–STAT signaling pathway and cytokine–cytokine receptor interaction, all of which are relevant to OA pathophysiology (Fig. 5f). Moreover, GO analysis revealed enrichment of terms including metal ion binding and cytoskeleton in gene bodies (Supplementary Fig. 9a). KEGG pathway analysis further demonstrated that the differentially methylated genes were associated with pathways such as regulation of actin cytoskeleton, the PI3K–Akt signaling pathway, metabolic pathways, the MAPK signaling pathway and the calcium signaling pathway (Supplementary Fig. 9b).

To understand how OSK regulates DNA methylation in OA, we examined the expression of DNMTs, the key enzymes modulating DNA methylation, within the DMM model. Immunohistochemistry results showed that the expression of DNA methyltransferase 3 alpha (DNMT3a), a chief enzyme responsible for adding methyl groups to DNA, significantly increased in the DMM-induced OA model compared to control mice (Fig. 5g, h). Crucially, post-OSK treatment, DNMT3a levels were noticeably declined (Fig. 5g, h), suggesting the capability of OSK to reverse or inhibit DNA methylation-driven cartilage aging.

To further dissect the epigenetic machinery, we examined the expression of Tet methylcytosine dioxygenase 1–3 (Tet1–3), a group of enzymes instrumental in DNA demethylation, within primary chondrocytes. Among these, *Tet2* emerged as a potential key critical player in the presence of OSK (Fig. 5i).

Collectively, our findings highlight that OSK could inhibit cartilage aging in OA by targeting key molecular players in the DNA methylation process.

### TET2 plays a crucial role in mediating the effects of OSK

To elucidate the integral role of TET2 in mediating the effects of OSK, we employed *Tet2*-specific small interfering RNA (siRNA) to suppress its expression. Intriguingly, post- *Tet2* silencing, the anti-inflammatory effects (Supplementary Fig. 10a) and the maintenance of chondrocyte function in the osteogenic environment (Supplementary Fig. 10b) by OSK were eliminated, underscoring the central role of *Tet2* in the OSK-driven epigenetic modulation.

**Fig. 6:**
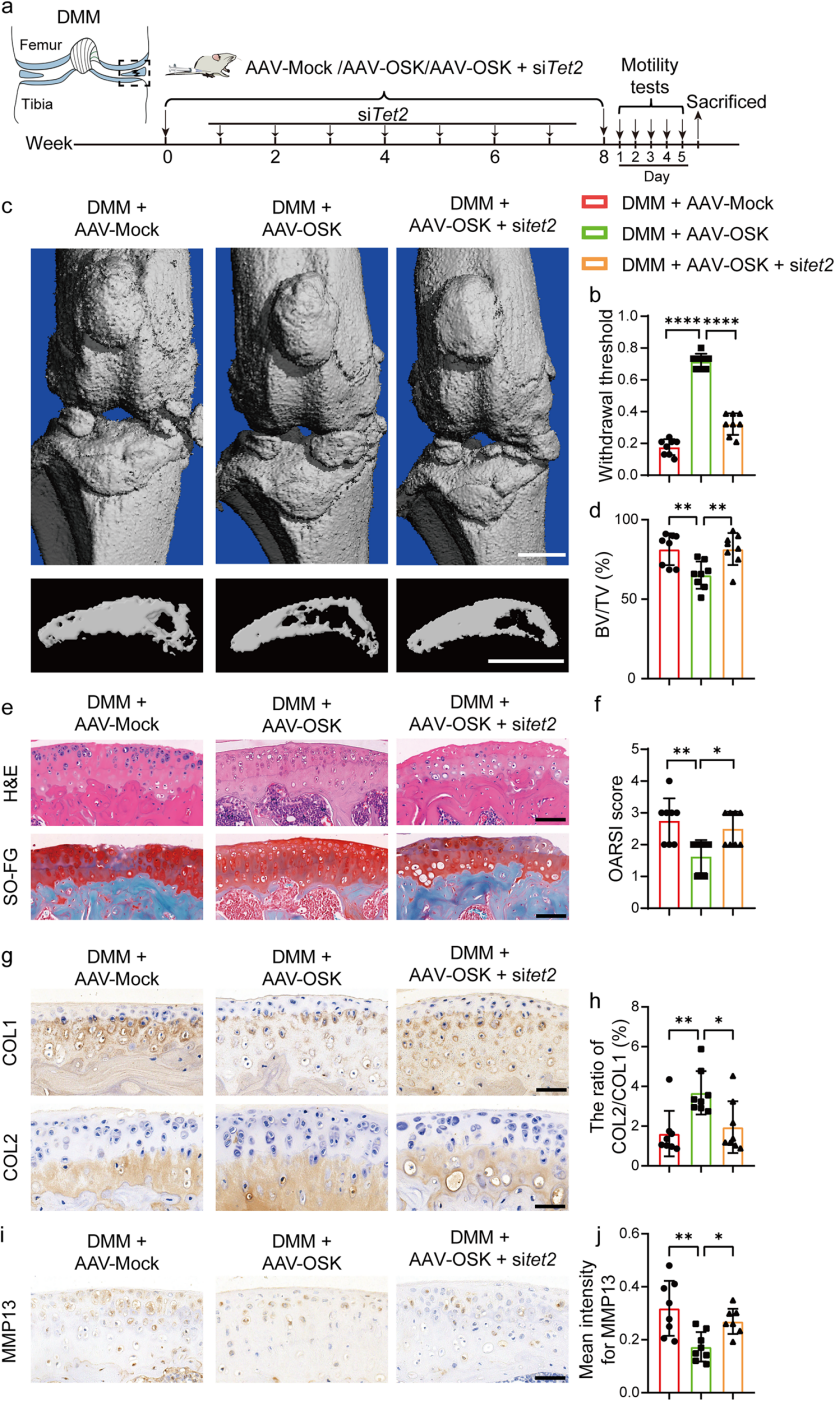
TET2 plays a crucial role in mediating the effects of OSK. **a** A diagram illustrating the timeline for DMM mouse modeling and intervention with OSK or si*Tet2*. **b** Pain measurement using the von Frey assay test. *n* = 8 per group. **c** Representative 3D reconstructed micro-CT images of the joint and sagittal views of tibia subchondral bone in treated mice 8 weeks after DMM surgery. Scale bar, 1 mm. **d** Quantitative analysis of the bone volume fraction of subchondral bone (BV/TV). *n* = 8 per group. **e** Representative images of H&E and SO-FG staining. Scale bar, 100 μm. **f** OARSI scores based on histological analysis of SO-FG staining. *n* = 8 per group. **g**, **h** Representative immunohistochemical staining of COL1 and COL2 (**g**) and quantification of the ratio of the positive area of COL2 to COL1 (**h**). Scale bar, 50 μm. *n* = 8 per group. **i**, **j** Representative immunohistochemical staining of MMP13 (**i**) and respective quantitative analysis of staining intensity (**j**). Scale bar, 50 μm. *n* = 8 per group. Data are presented as mean ± s.d. and analyzed using one-way ANOVA with Bonferroni post hoc test or nonparametric Kruskal–Wallis test (**f**). *P < 0.05, **P < 0.01, ***P < 0.001, ****P < 0.0001.

To assess the in vivo role of *Tet2*, a DMM-induced OA mice model was treated with AAV-Mock, AAV-OSK or AAV-OSK in combination with si*Tet2*. Following surgery, AAV injections were administered monthly, while si*Tet2* was delivered weekly for a total of 2 months (Fig. 6a). Following completion of the treatments, comprehensive assessments of joint pathology were performed. Behavioral tests, including grip strength and balance beam assays, revealed that mice receiving si*Tet2* in combination with OSK exhibited poorer performance compared to the AAV-OSK-treated group (Supplementary Fig. 11a). Consistently, *Tet2*-silenced mice displayed a lower pain threshold, indicative of heightened discomfort (Fig. 6b). Micro-CT analysis further showed that *Tet2* silencing markedly inhibited the OSK-mediated protection of joint surfaces while exacerbating subchondral bone formation (Fig. 6c, d). Histological evaluation using H&E and SO-FG staining revealed that *Tet2* silencing attenuated the OSK-induced mitigation of cartilage degeneration characteristic of OA (Fig. 6e, f). Immunohistochemistry confirmed that *Tet2* knockdown suppressed OSK-promoted expression of cartilage synthetic markers ACAN (Supplementary Fig. 11b, c). Moreover, TET2 silencing decreased the ratio of COL2 to COL1 ratio in repair tissues, indicating a shift toward chondrofibrosis (Fig. 6g, h). In parallel, the inhibitory effects of OSK on catabolic and hypertrophic markers, such as MMP13 (Fig. 6i, j) and COLX (Supplementary Fig. 11d, e), were substantially reduced.

Collectively, these results demonstrate that *Tet2* is critical for OSK-mediated protection and anabolic remodeling in DMM-induced OA.

### OSK-mediated epigenetic modulation promotes fibrocartilage hyalinization

**Fig. 7:**
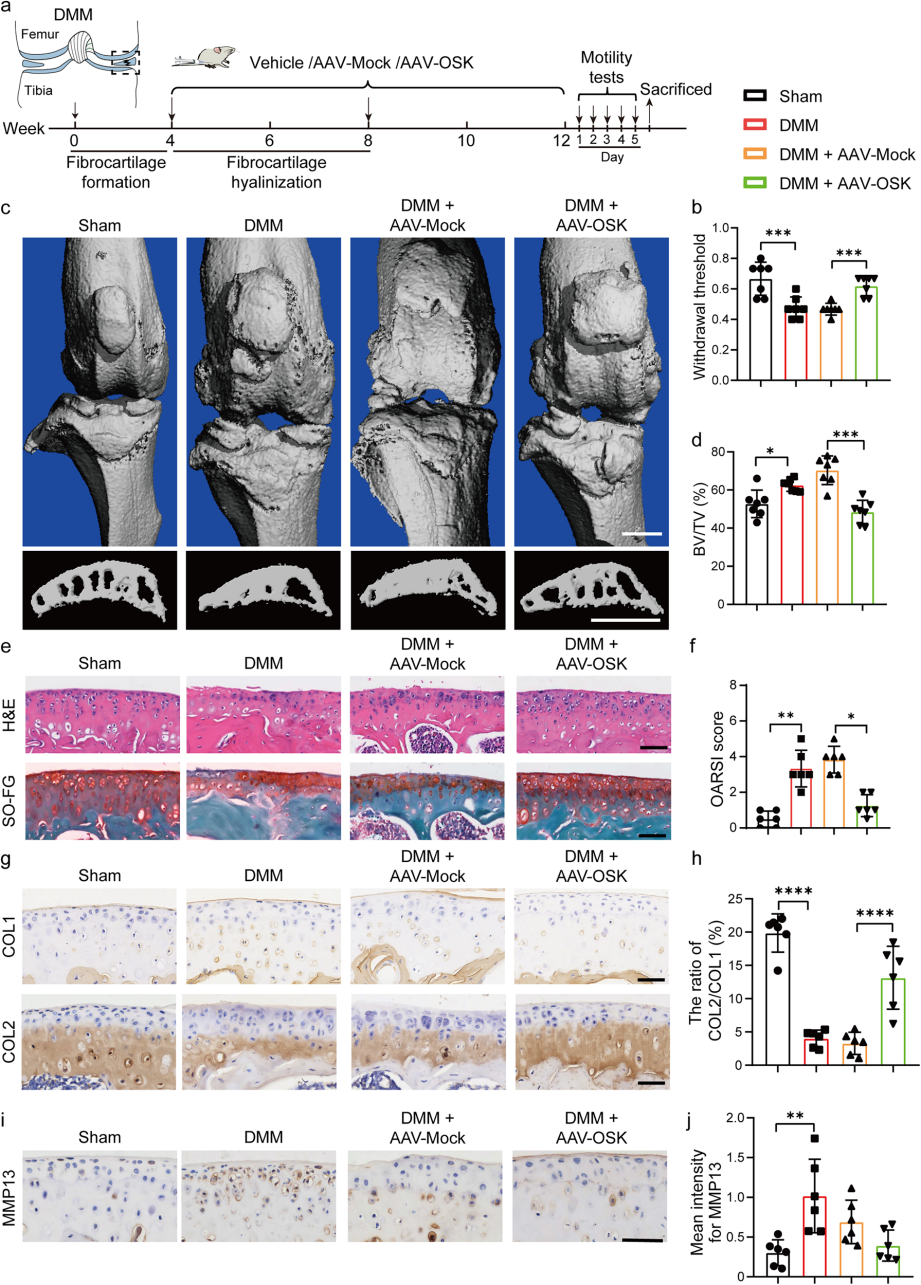
OSK-mediated epigenetic modulation promotes fibrocartilage hyalinization. **a** A diagram illustrating the timeline for the post-onset OA mice model and intervention. **b** Pain measurement using the von Frey assay test. *n* = 6 per group. **c** Representative 3D reconstructed micro-CT images of the joint and sagittal views of tibia subchondral bone in treated mice 12 weeks after DMM surgery. Scale bar, 1 mm. **d** Quantitative analysis of bone volume fraction of subchondral bone (BV/TV). *n* = 6 per group. **e** Representative images of H&E and SO-FG staining. Scale bar, 100 μm. **f** OARSI scores based on histological analysis of SO-FG staining. *n* = 6 per group. **g**, **h** Representative immunohistochemical staining of COL1 and COL2 (**g**) and quantification of the ratio of the positive area of COL2 to COL1 (**h**). Scale bar, 50 μm. *n* = 6 per group. **i**, **j** Representative immunohistochemical staining of MMP13 (**i**) and quantitative analysis of staining intensity (**j**). Scale bar, 50 μm. *n* = 6 per group. Data are presented as mean ± s.d. and analyzed using one-way ANOVA with Bonferroni post hoc test or nonparametric Kruskal–Wallis test (**f**). *P < 0.05, **P < 0.01, ***P < 0.001, ****P < 0.0001.

To investigate the potential of OSK in promoting the hyalinization of fibrocartilage in vivo, we utilized a DMM mouse model, which simulates cartilage injury and the subsequent formation of fibrocartilage, often observed as an end-stage pathology in OA. At 4 weeks after surgery, once fibrocartilage had formed, we performed an intra-articular injection of either AAV-Mock or AAV-OSK at 4- and 8-weeks postsurgery (Fig. 7a). Initial pain and movement assessments conducted 4 weeks after model initiation confirmed a lower paw withdrawal threshold (Supplementary Fig. 12a) and worsened motor capabilities (Supplementary Fig. 12b) in the DMM mice, indicating the successful establishment of the OA model.

At 8 weeks after the initiation of OSK treatment, a comprehensive evaluation of the joint pathology was undertaken. Grip strength test and balance beam test revealed that OSK-treated mice exhibited superior behavioral assessments compared to their OA counterparts treated with AAV-Mock (Supplementary Fig. 12c). In addition, OSK-treated mice demonstrated an increased pain threshold, indicative of reduced OA-associated discomfort (Fig. 7b). Micro-CT imaging revealed that the cartilage erosion and escalated subchondral bone formation evident in the OA group were marked reversed by OSK treatment (Fig. 7c, d).

Next, H&E staining and SO-FG staining revealed that OSK treatment significantly mitigated the cartilage deterioration typically seen in OA (Fig. 7e, f). Immunohistochemistry analyses further revealed that OSK facilitated the expression of anabolic cartilage markers ACAN (Supplementary Fig. 12d, e). Notably, the ratio of COL2 to COL1 within the cartilage region was significantly increased following OSK treatment, suggesting a shift toward hyaline cartilage restoration (Fig. 7g, h). Moreover, this rejuvenative effect was paired with a suppression of catabolic and hypertrophic markers, including MMP13 (Fig. 7i, j) and COLX (Supplementary Fig. 12f, g), respectively.

In summary, these results indicate the promising effect of OSK in transforming fibrocartilage to hyaline cartilage in situ.

## Discussion

**Fig. 8:**
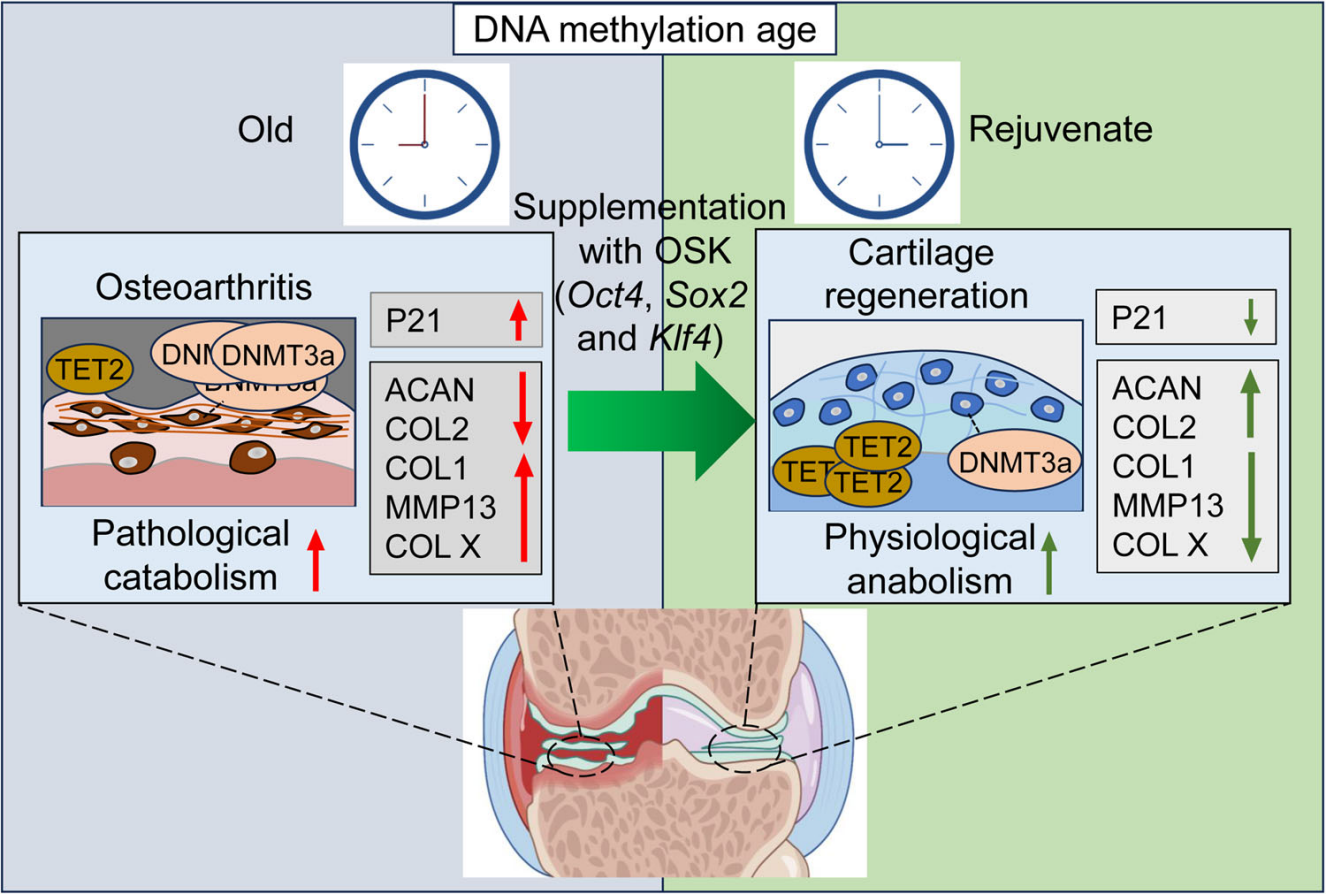
Schematic diagram. OA development is closely linked to aging, characterized by changes in DNA methylation that reflect the senescent state of chondrocytes. In the OA condition, the expression of senescence markers P21 and DNMT3a were elevated. Concurrently, the pathological catabolism of articular chondrocytes increased, resulting in extracellular matrix degeneration and the formation of fibrocartilage. Supplementation with exogenous OSK factors reversed cellular senescence, altered DNA methylation patterns and enhanced physiological anabolism in chondrocytes. These effects promoted fibrocartilage hyalinization and contributed to the prevention of OA, ultimately aiding in the restoration of cartilage homeostasis.

OA, the most prevalent degenerative joint disease, presents with a range of clinical symptoms, including cartilage degradation, joint pain, stiffness and subsequent loss of function. Current therapeutic approaches primarily aim to alleviate these symptoms, yet there is an urgent need for innovations capable of halting or potentially reversing OA progression. Interventions that solely focus on a single aspect, such as biomechanics or inflammation, may not adequately address the multifactorial nature of OA. Recent advances in epigenetics have prompted us to explore the potential of OSK, factors for partial reprogramming, offering a new perspective for OA from an epigenetic angle (Fig. 8).

In this study, we first ascertained that OSK could be effectively expressed in chondrocytes, where it exerts protective effects against inflammation, apoptosis and degeneration. Second, we evaluated the regenerative potential of OSK within established murine OA models, such as the DMM and ACLT surgeries. Third, we investigated the influence of OSK on the epigenetic landscape of OA, focusing particularly on key DNA methylation enzymes and aging. The evidence has demonstrated that OSK confers protection to chondrocytes and has exhibited a notable capacity to rejuvenate cartilage aging and ameliorate the OA phenotype.

The cellular reprogramming machinery, particularly the transformative potential of OSK, offers an intriguing possibility for OA treatment. By inducing a pluripotent-like state, these transcription factors can potentially rejuvenate the self-renewal and differentiation capacities of specialized cells^47^. Partial reprogramming with OSK provides a safer and more precise alternative to OSKM, as omission of c-Myc markedly lowers oncogenic burden while preserving epigenetic rejuvenation capacity. In retinal ganglion cells, sustained AAV-OSK expression restores youthful DNA methylation patterns, without triggering proliferation or tumor formation even after 10–18 months of continuous expression^23^. However, as systemic effects on other organs remain underexplored, we have confined the discussion of safety advantages of OSK to retinal and similar localized applications, emphasizing the need for broader organ-specific evaluations in future studies. Mechanistically, c-Myc is dispensable for partial reprogramming, as it primarily counteracts an OSK-induced proliferation pause mediated by elevated p16/p21 and reduced endogenous MYC^48^, whereas chronic MYC activation heightens metabolic and transcriptional stress to promote tumorigenesis^49^. The implications for OA are substantial: OSK might safeguards chondrocyte identity by suppressing maladaptive hypertrophic maturation and osteogenic-like differentiation^50,51^, without fully reverting chondrocytes to a stem cell state. The local administration of viral vectors overexpressing OSK factors hold promise for targeted partial reprogramming within the joint cavity, promoting tissue regeneration and attenuating OA progression. This approach could restore the balance between cartilage degradation and repair, leading to improved joint functionality. Moreover, localized delivery within the joint cavity can potentially amplify the therapeutic efficacy while minimizing systemic adverse effects. However, before clinical extrapolation, it is essential to determine the optimal factor dosage, assess the stability and longevity of reprogrammed cells and anticipate long-term safety profiles.

Our findings also highlight the ability of OSK to mitigate the loss of anabolic factors and regulate catabolic processes in chondrocytes, which are central to OA progression. Notably, our study contrasts with other reprogramming strategies, such as lin-28 homolog A (Lin28a), which have been shown to restore the stemness of chondrocytes (high expression of Nanog) and promote chondrocyte regeneration^52^. Meanwhile, their findings of decreased cartilage degradation and increased osteophyte volume also suggest that Lin28a reprograms chondrocytes into progenitor cells that can form osteophytes^52^. Unlike Lin28a, OSK does not induce a complete reversion to a stem cell state but instead facilitates a controlled rejuvenation of cartilage tissue. This is particularly important in the context of OA, where the active proliferation of chondrocytes in response to environmental stimuli often leads to changes in cellular function, metabolism, and cytoskeletal organization, contributing to increased expression of COL1 and eventual hyaline cartilage fibrosis^53^.

A particularly intriguing finding of our study is the interplay between OSK and the epigenetic clock, specifically DNA methylation. DNA methylation is a critical player in the aging process of cartilage, with recent studies revealing DNA methylation changes in many genes related to OA, including the transcription factor SOX9, ECM proteins, anabolic factors COL2 and ACAN, and matrix degradation protease MMP13^54^. In this study, we confirmed that OSK can reduce the loss of anabolic factors and control catabolic trends in chondrocytes, important for OA progression. We also investigated the epigenetic mechanisms related to abnormal DNA methylation by examining DNA methyltransferase expression. Previous research indicates that DNMT3b expression decreases at OA onset, while its overexpression protects cartilage in a surgical mouse model of OA^55^. The levels of DNMT1 and DNMT3a significantly increased in cartilage of OA patients and DMM mice^16^, while the level of DNMT3b decreased slightly, which aligns with our findings. Intriguingly, TET enzymes are involved in demethylation, and inhibiting TET1 can prevent the development of OA^56^. Our investigation revealed that the OSK intervention has the potential to rejuvenate senescent cells. However, the limited sample size in our study precludes the attainment of statistical significance. It is recommended that future research endeavors enhance the sample size for the detection of DNA methylation age. In addition, the OSK intervention was observed to upregulate TET2 expression in chondrocytes. The attenuation of the protective effects of OSK on cartilage following TET2 silencing suggests that TET2 plays a mediating role in the mechanism of OSK action. Although the use of OSK factors for partial reprogramming in OA has demonstrated considerable potential, several challenges remain to be addressed. First, the precise dosage and timing of factor administration must be optimized to ensure efficient reprogramming without inducing undesirable effects. Nevertheless, the absence of extensive longitudinal studies on OSK treatment raises potential safety concerns regarding its prolonged expression. Although persistent OSKM expression has been linked to tumorigenic risks^57^, the long-term implications of OSK expression remain ambiguous. Future studies should employ inducible and lineage-tracing systems in relevant animal models to perform comprehensive biodistribution and oncogenicity assessments, including long-term monitoring of vector persistence across major organs and detailed histopathological analyses to detect any aberrant morphological changes or tumor formation. In addition, the efficacy of our interventions has only been demonstrated in murine models. Translating these successes to larger mammals, and eventually to humans, will require refined strategies, possibly leveraging the strengths of AAVs, vesicle encapsulation or mRNA-based therapies^58^. We have confirmed the feasibility of AAV-mediated delivery as AAVs offer tissue-specific tropism, efficient transduction, low immunogenicity, episomal persistence and sustained gene expression, positioning them as a leading tool for clinical gene therapy. However, several limitations persist, including restricted transduction in certain tissues, suboptimal organ specificity, pre-existing immunity to capsids and dose-dependent toxicity. Therefore, further approaches are required for validation and comparative evaluation^59^. Furthermore, while the epigenetic landscape offers considerable potential, the intricacies of DNA methylation in OA remains enigmatic. The exact mechanism by which OSK exerts its effects, particularly via TET2 and its demethylation capabilities, requires further investigation.

Our study into cellular reprogramming and epigenetics for OA represents an illustration of the evolving frontiers of regenerative medicine. By delving deep into the transformative potential of transcription factor-driven reprogramming, this study aims to contribute to a redefinition of OA therapeutic strategies. The findings of this research may provide insight into the future prospects of epigenetic-based interventions for OA and pave the way for personalized and regenerative medicine approaches in the management of this prevalent musculoskeletal disorder.

In conclusion, our study provides compelling evidence that the OSK factors hold significant promise in the therapeutic landscape of OA. By modulating critical epigenetic markers and preventing chondrocyte dedifferentiation, OSK not only preserves the chondrocyte phenotype but also promotes cartilage regeneration. Our findings demonstrate that OSK administration, even after the onset of OA, can significantly reverse the pathogenesis of the disease, underscoring its potential for clinical application in OA therapy. Moreover, our investigation into the epigenetic mechanisms underlying the therapeutic effects of OSK revealed its capacity to modulate DNA methylation by upregulating TET2. Collectively, the ability of OSK to influence both the molecular and structural aspects of cartilage underscores its value as a promising strategy for the treatment of this debilitating joint disease, particularly in addressing the challenges posed by hyaline cartilage fibrosis.

## Supplementary information

Supplementary Information

## Data Availability

The data and materials are available from corresponding authors on reasonable request.
