# Supplementary material for: Local delivery of OSK factors enables partial cellular reprogramming to mitigate osteoarthritis and cartilage fibrosis
Source: Exp Mol Med. 2026 Mar 5;58(3):782–97. doi: 10.1038/s12276-026-01662-x (PMC13049178; doi:10.1038/s12276-026-01662-x)
Supplement: Supplementary file 1 — Supplementary Information [file 12276_2026_1662_MOESM1_ESM.docx]

**Supplementary Information**

**Local Delivery of OSK Factors Enables Partial Cellular Reprogramming to Mitigate Osteoarthritis and Cartilage Fibrosis**

Yi-Wei Liu^1,2^, Jing-Tao Zou^1,2^, Jiang-Shan Gong^1,2^, Ling Jin^1,2^, Jia Cao^1,2,4^, Ze-Hui He^1,2^, Yu-Xuan Qian^1,2^, Xin Wang^1,2^, Mei-Dan Wan^3^, Xin-Yue Hu^1,2,5^, Chun-Gu Hong^1,2^, Wei Du^1,2,4^, Chun-Yuan Chen^1,2,4^, Hong-Ji Liu^1,2^*, Hui Xie^1,2,4^*, Zhen-Xing Wang^1,2,4^*

Affiliations

1 Department of Orthopedics, Movement System Injury and Repair Research Center, Xiangya Hospital, Central South University, Changsha, Hunan 410008, China

2 Hunan Key Laboratory of Angmedicine, Changsha, Hunan 410008, China

3 Department of Neurology, Xiangya Hospital, Central South University, Changsha, Hunan 410008, China

4 National Clinical Research Center for Geriatric Disorders (Xiangya Hospital), Changsha, Hunan 410008, China

5 Department of Respiratory Medicine, Xiangya Hospital, Central South University, Changsha, Hunan 410008, China

* Correspondence authors: huixie@csu.edu.cn (Dr. Hui Xie), wangzx@csu.edu.cn (Dr. Zhen-Xing Wang), liuhongji@hnu.edu.cn (Dr. Hong-Ji Liu).

**
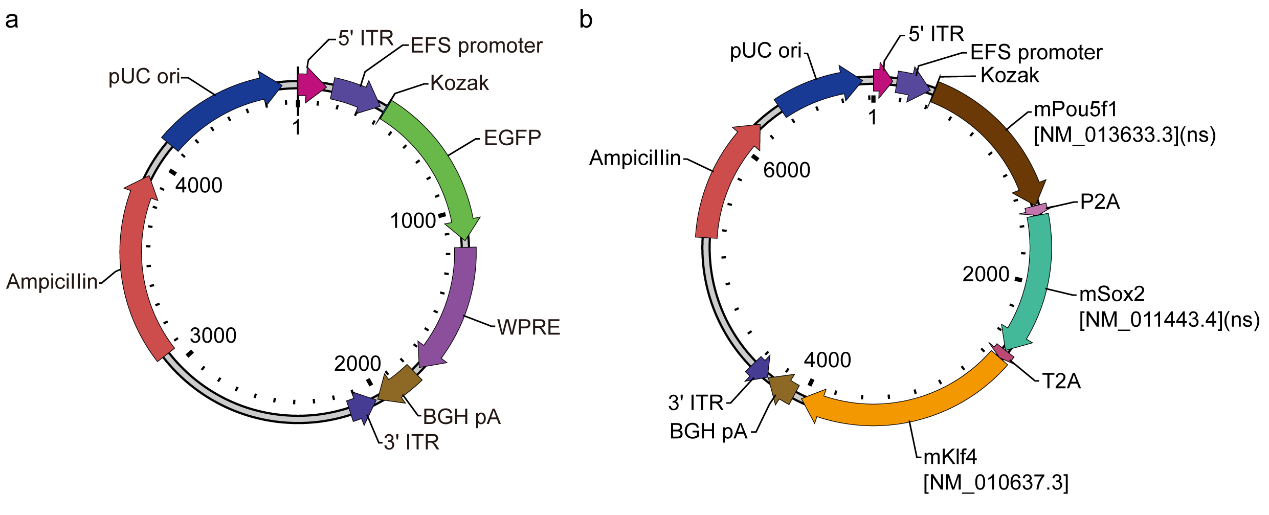
**

**Supplementary Fig. 1. Construction of a vector carrying OSK.** (a-b) Construction of the control adeno-associated virus vector (AAV-Mock; a) and adeno-associated virus harboring OSK (AAV-OSK; b).


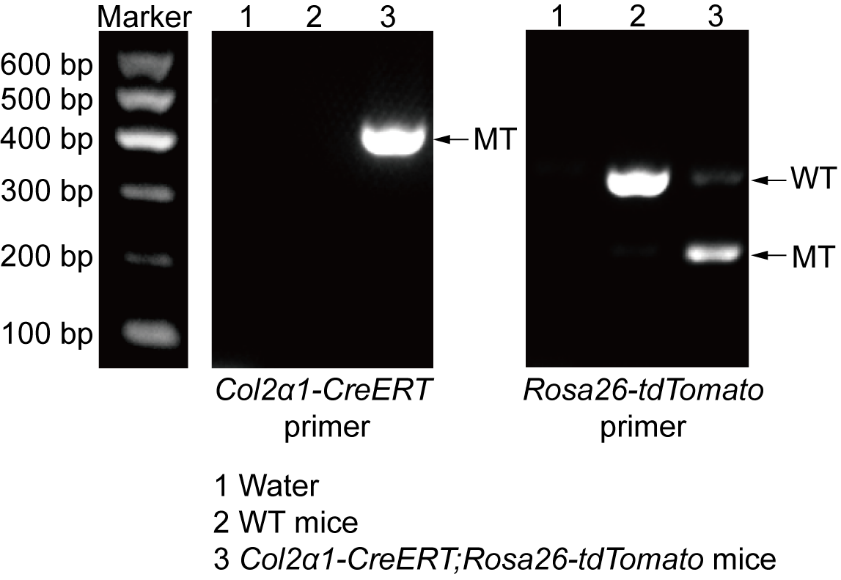


**Supplementary Fig. 2. Genotyping identification of *Col2α1-CreERT;Rosa26-tdTomato* reporter mice.** PCR diagram for the identification of the *Col2α1-CreERT* gene and *Rosa26-tdTomato* gene. WT: wild type. MT: mutant.


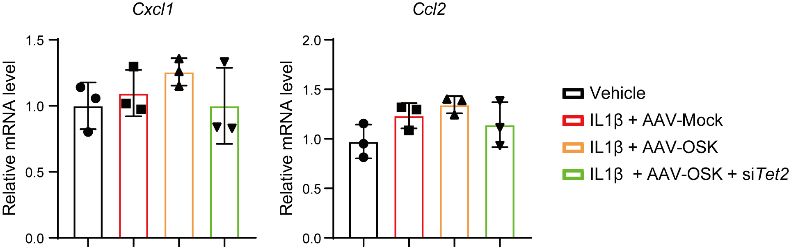


**Supplementary Fig. 3.** **OSK exhibits anti-inflammatory and anti-apoptotic effects in chondrocytes.** qRT-PCR analysis of *Cxcl1* and *Ccl2* in ATDC5 cell line under inflammatory environment induced by IL-1β for 24 h, after treated with vehicle, AAV-Mock, or AAV-OSK for 3 days.


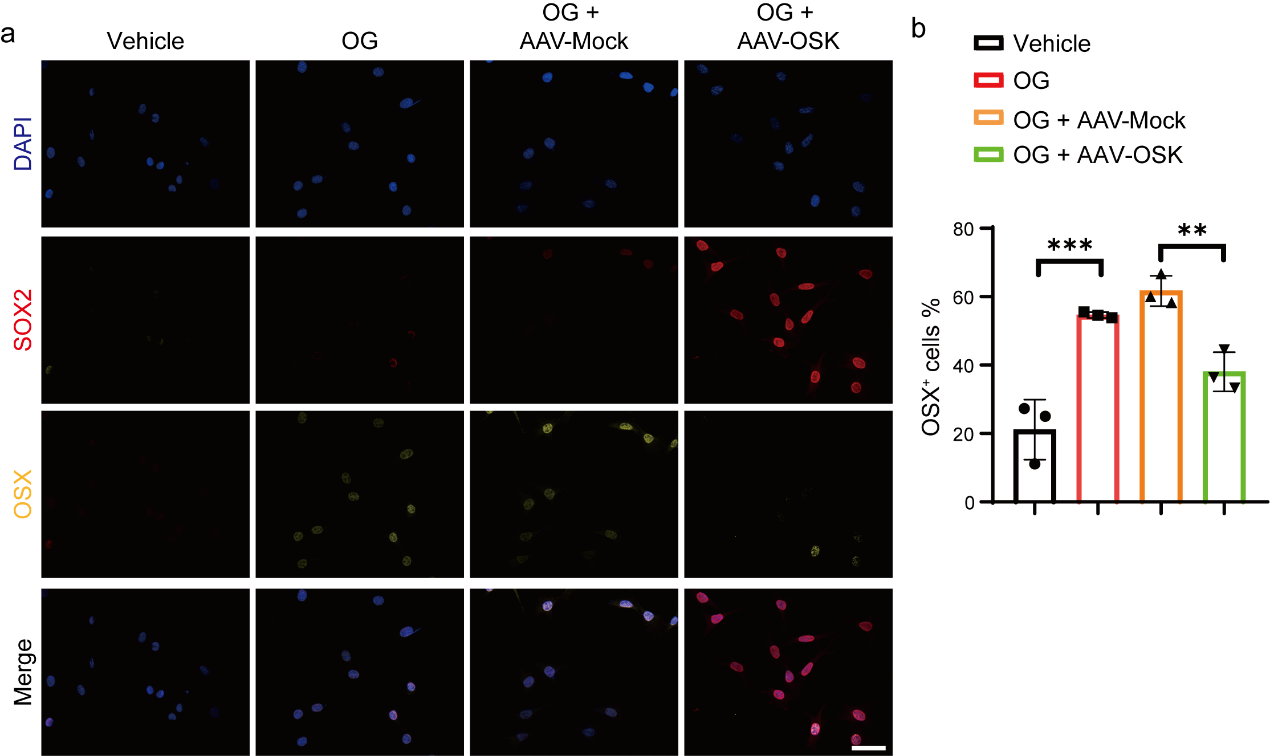
 **Supplementary Fig. 4. OSK counteract osteogenic transition and preserve chondrocyte phenotype.** (a and b) ICC staining of SOX2 and OSX in ATDC5 cells under osteogenic conditions (b) and the percentage of OSX positive cells (c). n = 3 per group. Scale bar: 50 μm.


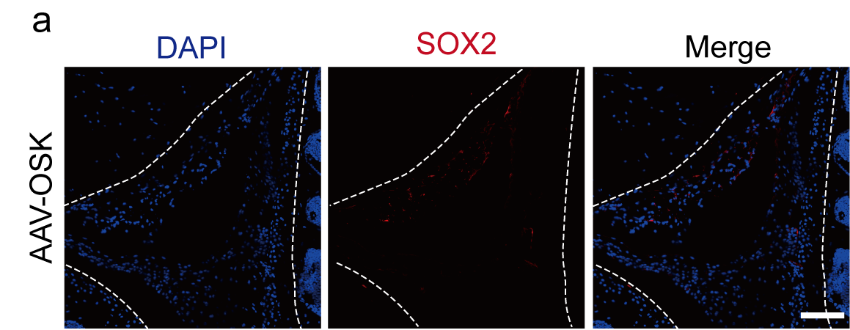


**Supplementary Fig. 5. OSK expresses in chondrocytes without hyperproliferation** (a) Immunofluorescence staining of SOX2 in synovial membrane from mice treated with AAV-OSK. Scale bar: 100 μm. White dash line indicated the synovial region.

**Supplementary Fig. 6. Behavioral analysis of DMM-induced OA mice model.** (a) Movement of mice was evaluated by the grip strength test and balance beam test for five consecutive days. n = 8 per group, three repeats for each mouse. Asterisks indicate significance differences between the AAV-OSK and AAV-Mock groups. (b-e) Representative immunohistochemical staining of ACAN (b) and COLX (d), and respective quantitative analysis of staining intensity (c and e). Scale bar: 50 μm. n = 8 per group. Data were presented as mean ± SD and analyzed using two-way ANOVA with Bonferroni test (a) or one-way ANOVA with Bonferroni post hoc test (c and e). * *P* < 0.05, ** *P* < 0.01, *** *P* < 0.001, **** *P* < 0.0001.


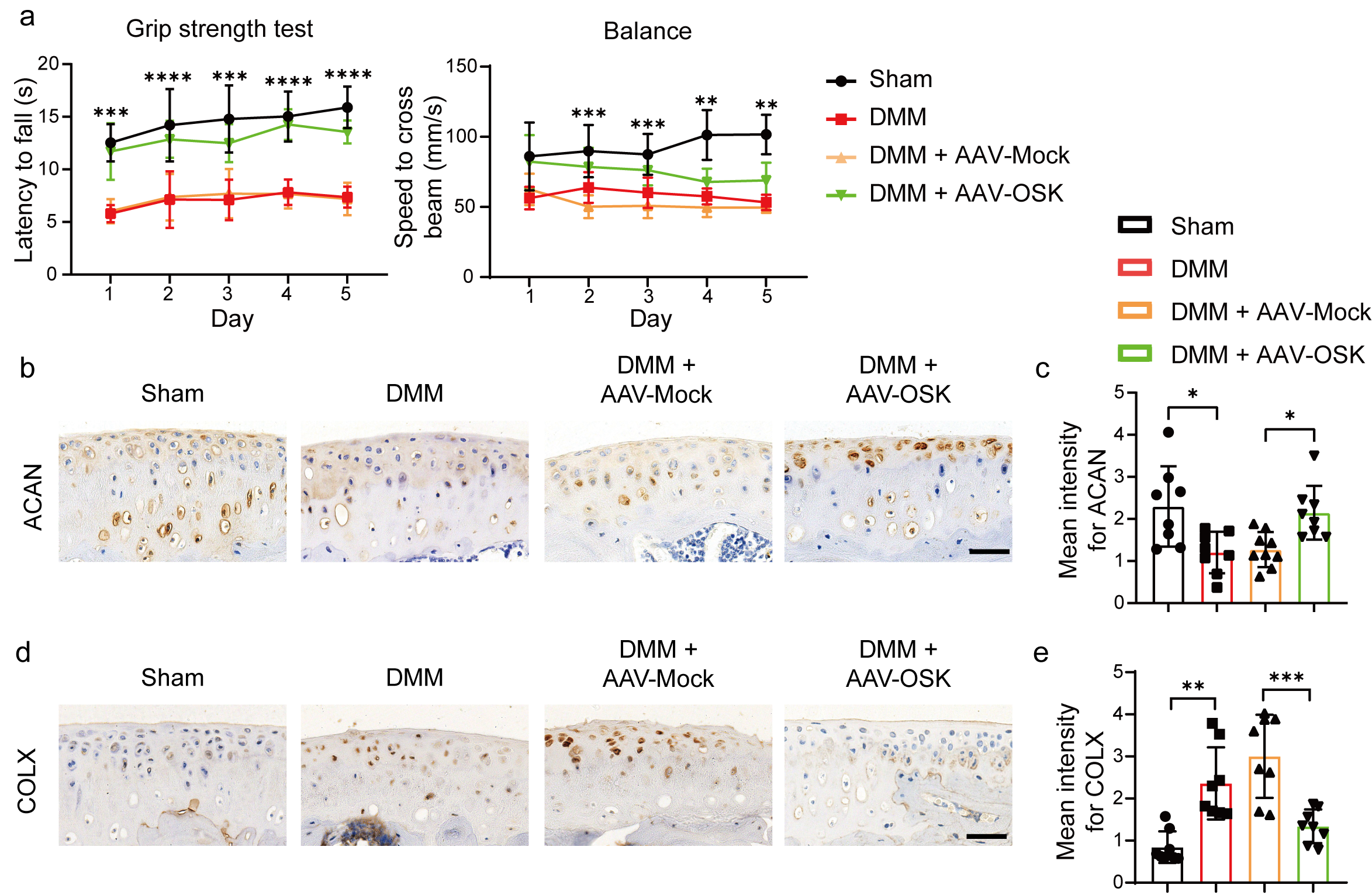


**Supplementary Fig. 7. Amelioration of ACLT-induced OA via OSK expression.** (a) Diagram illustrating the timeline for ACLT mouse modeling and intervention with OSK. (b) Pain measurement using the von Frey assay test. n = 6 per group. (c) Representative 3D reconstructed micro-CT images of the joint and sagittal views of tibia subchondral bone in treated mice 8 weeks after ACLT surgery. Scale bar: 1 mm. (d) Quantitative analysis of bone volume fraction of subchondral bone (BV/TV). n = 6 per group. (e) Representative images of H&E and SO-FG staining. Scale bar: 100 μm. (f) OARSI scores based on histological analysis of SO-FG staining. n = 6 per group. (g-h) Representative immunohistochemical staining of COL1 and COL2 (g), and quantification of the ratio of the positive area of COL2 to COL1 (h). Scale bar: 50 μm. n = 6 per group. (i-j) Representative immunohistochemical staining of MMP13 (i) and quantitative analysis of staining intensity (j). Scale bar: 50 μm. n = 6 per group. Data were presented as mean ± SD and analyzed using one-way ANOVA with Bonferroni post hoc test or nonparametric Kruskal-Wallis test (f). * *P* < 0.05, ** *P* < 0.01, *** *P* < 0.001, **** *P* < 0.0001.


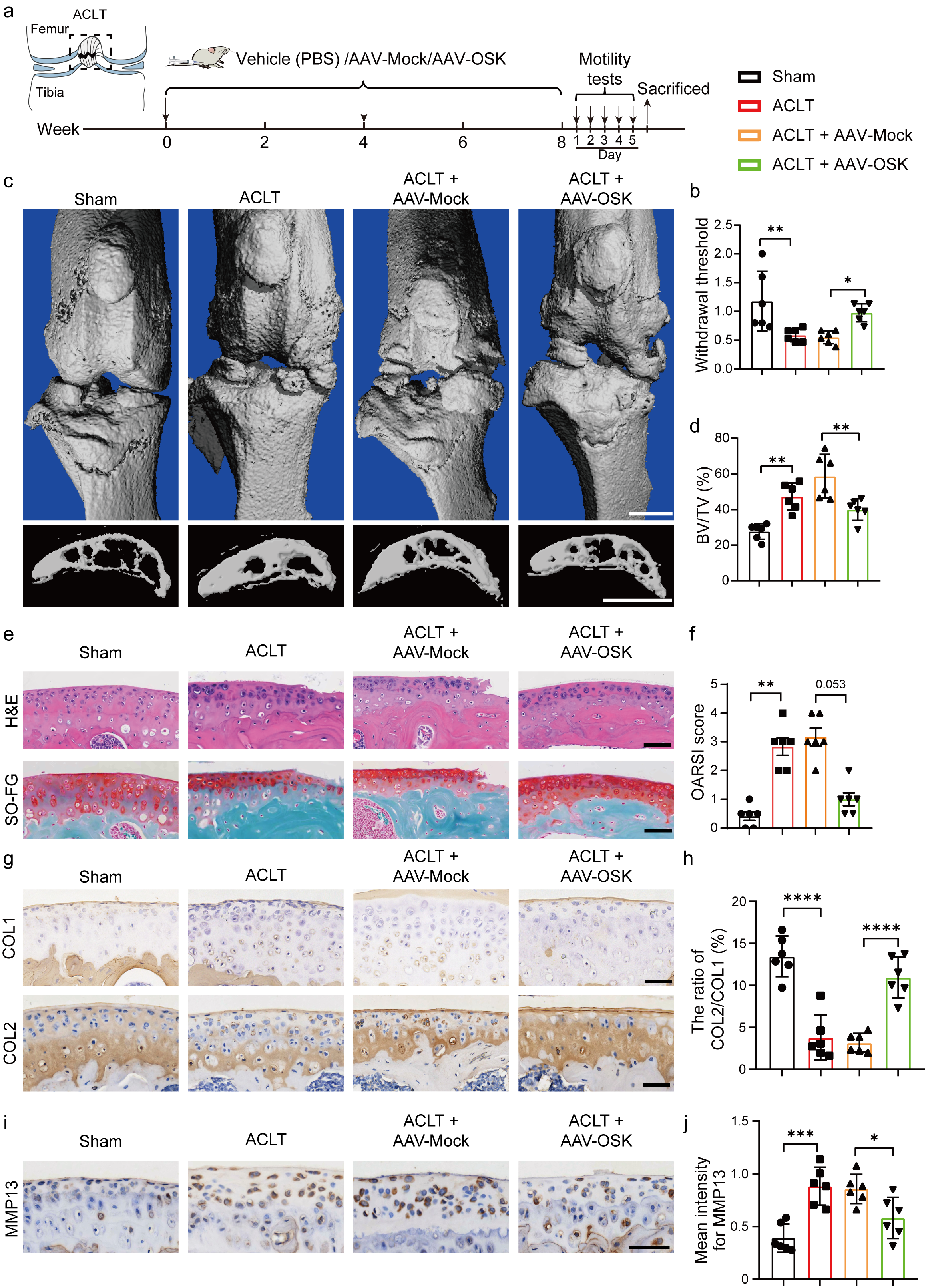

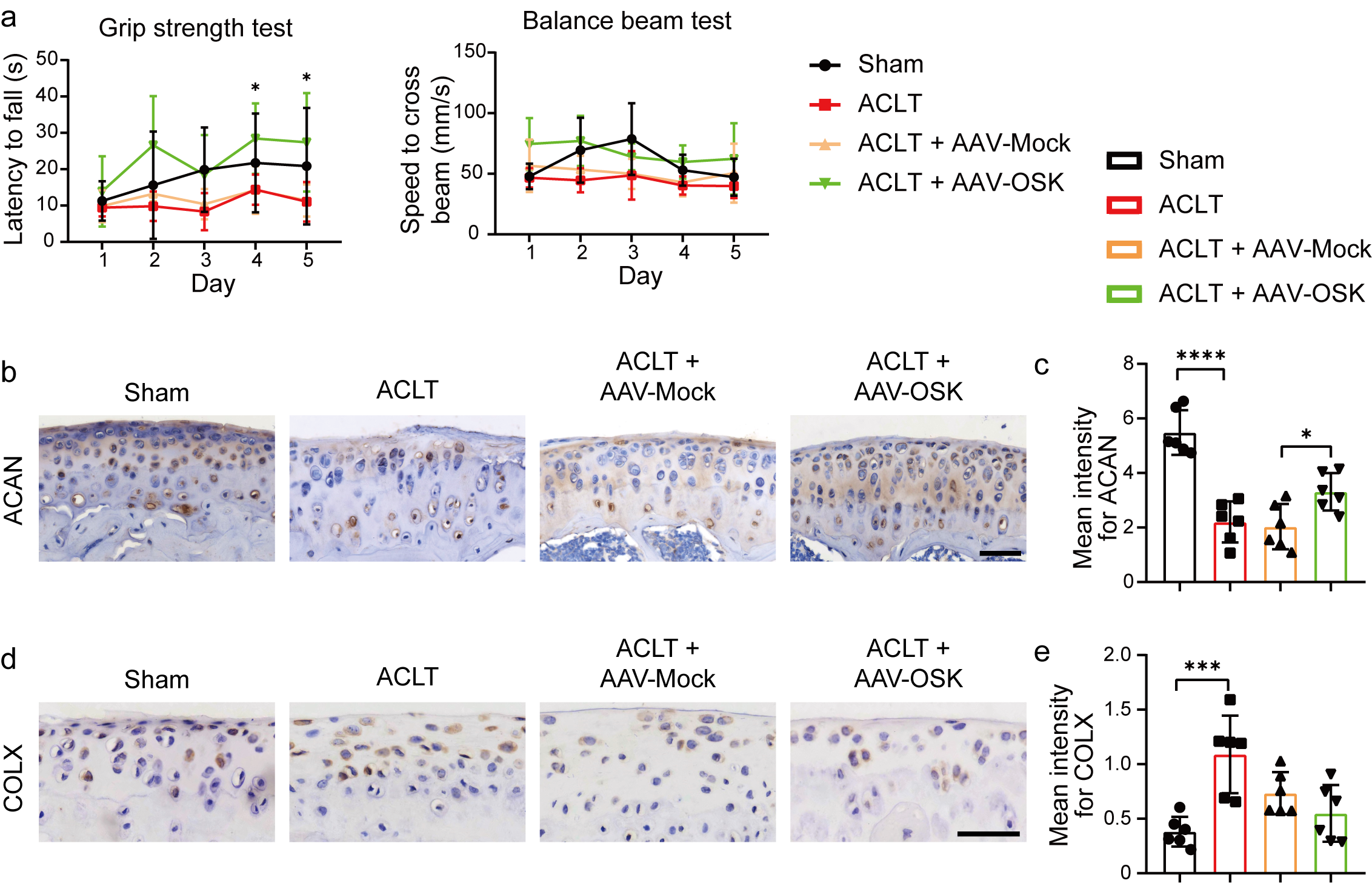


**Supplementary Fig. 8. Behavioral analysis of ACLT-induced OA mice model.** (a) Movement of mice was evaluated by the grip strength test and balance beam test for five consecutive days. n = 5-6 per group, three repeats for each mouse. Asterisks indicate significance differences between the AAV-OSK and AAV-Mock groups. (b-e) Representative immunohistochemical staining of ACAN (b) and COLX (d), and respective quantitative analysis of staining intensity (c and e). Scale bar: 50 μm. n = 6 per group. Data were presented as mean ± SD and analyzed using two-way ANOVA with Bonferroni test (a) or one-way ANOVA with Bonferroni post hoc test (c and e). * *P* < 0.05, *** *P* < 0.001, **** *P* < 0.0001.


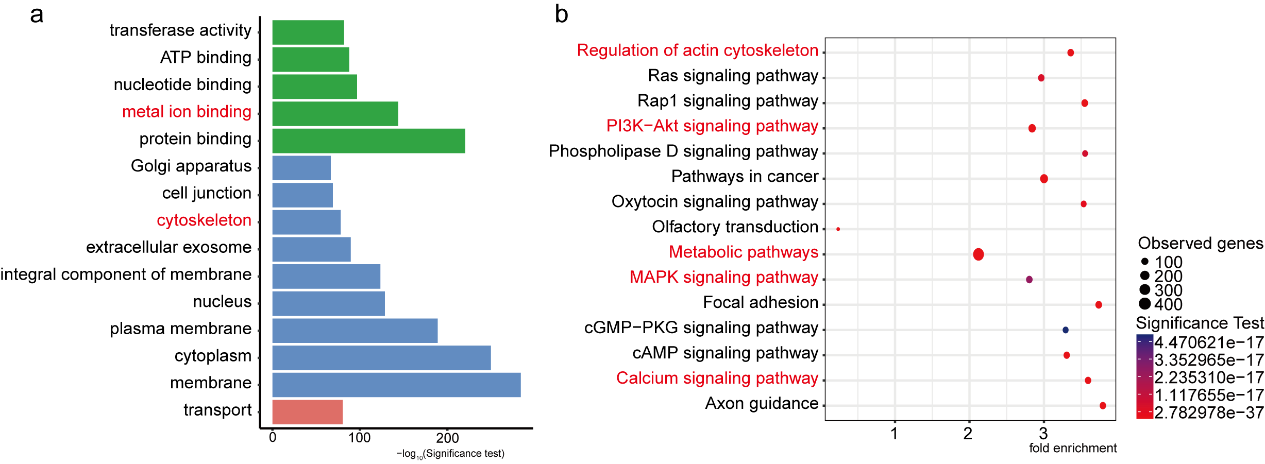


**Supplementary Fig. 9. OSK attenuate OA-driven epigenetic aging via DNA methylation modulation.** (a-b) Top enriched terms of biological processes (a) and top enrichment KEGG pathways (b) that were significantly altered gene bodies among AAV-Mock and AAV-OSK treated DMM mice.


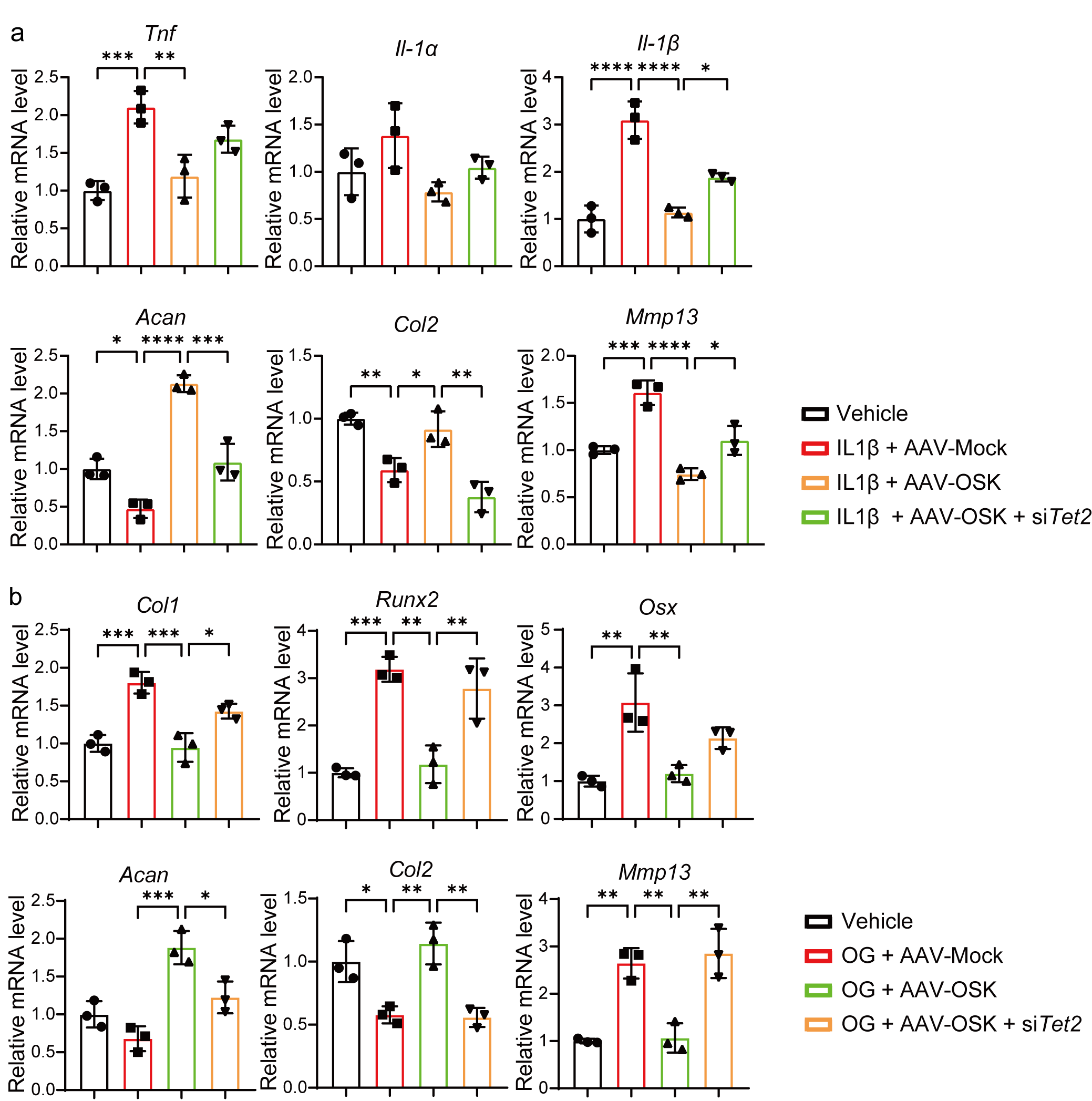
**Supplementary Fig. 10.** **TET2 Plays a Crucial Role in Mediating the Effects of OSK.** (a) qRT-PCR analysis of inflammatory gene (*Tnf*, *Il-1α* and *Il-1β*) and metabolic genes (*Acan*, *Col2* and *Mmp13*) in ATDC5 cells under inflammatory conditions with indicated treatments. n = 3 per group. (b) qRT-PCR analysis of osteogenic genes (*Col1*, *Runx2* and *Osx*) and metabolic genes (*Acan*, *Col2* and *Mmp13*) in ATDC5 cells under osteogenic conditions with indicated treatments. n = 3 per group. Data were presented as mean ± SD and analyzed using one-way ANOVA with Bonferroni post hoc test. * *P* < 0.05, ** *P* < 0.01, *** *P* < 0.001, **** *P* < 0.0001.

**
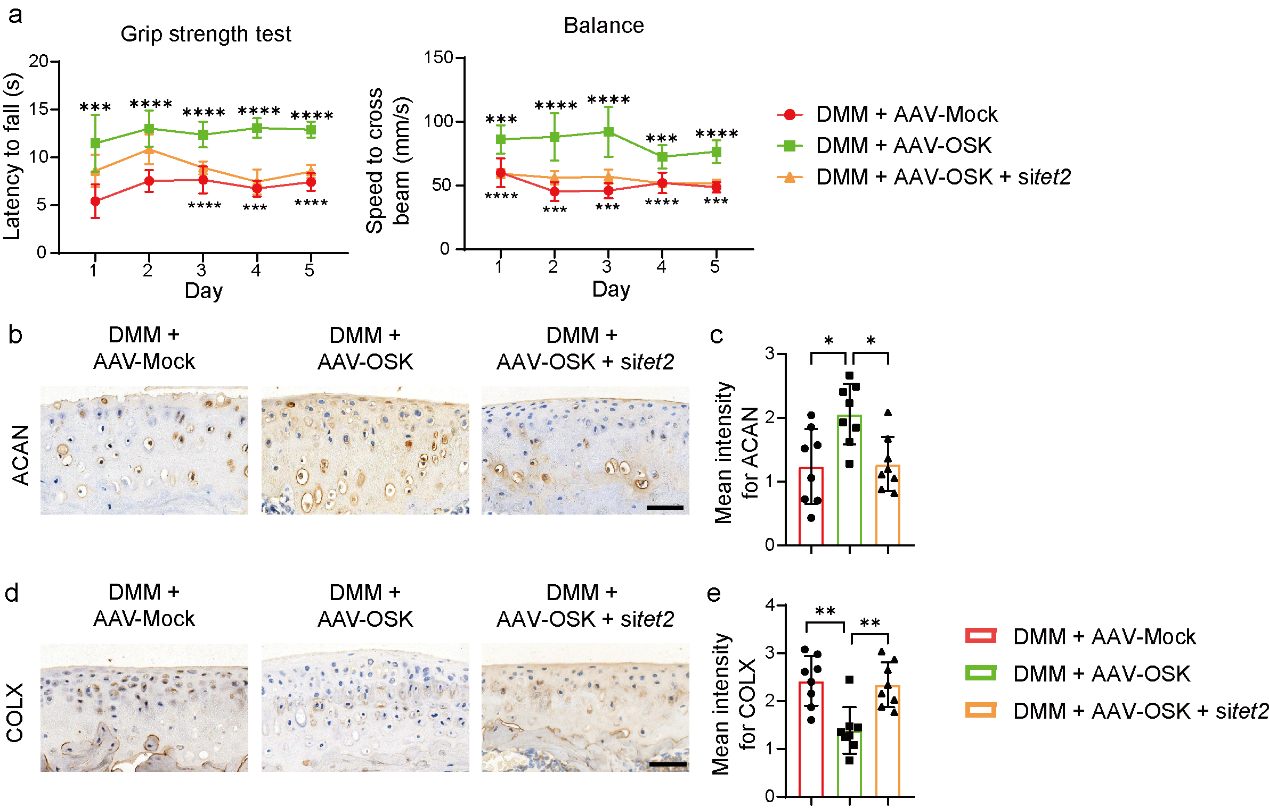
Supplementary Fig. 11. Behavioral analysis of DMM-induced OA mice model.** (a) Movement of mice was evaluated by the grip strength test and balance beam test for five consecutive days. n = 8 per group, three repeats for each mouse. Asterisks indicate significance differences between the AAV-OSK and AAV-Mock groups (upper) or AAV-OSK + si*tet2* and AAV-OSK groups (lower). (b-e) Representative immunohistochemical staining of ACAN (b) and COLX (d), and respective quantitative analysis of staining intensity (c and e). Scale bar: 50 μm. n = 8 per group. Data were presented as mean ± SD and analyzed using two-way ANOVA with Bonferroni test (a) or one-way ANOVA with Bonferroni post hoc test (c and e). * *P* < 0.05, ** P < 0.01, *** *P* < 0.001, **** P < 0.0001.


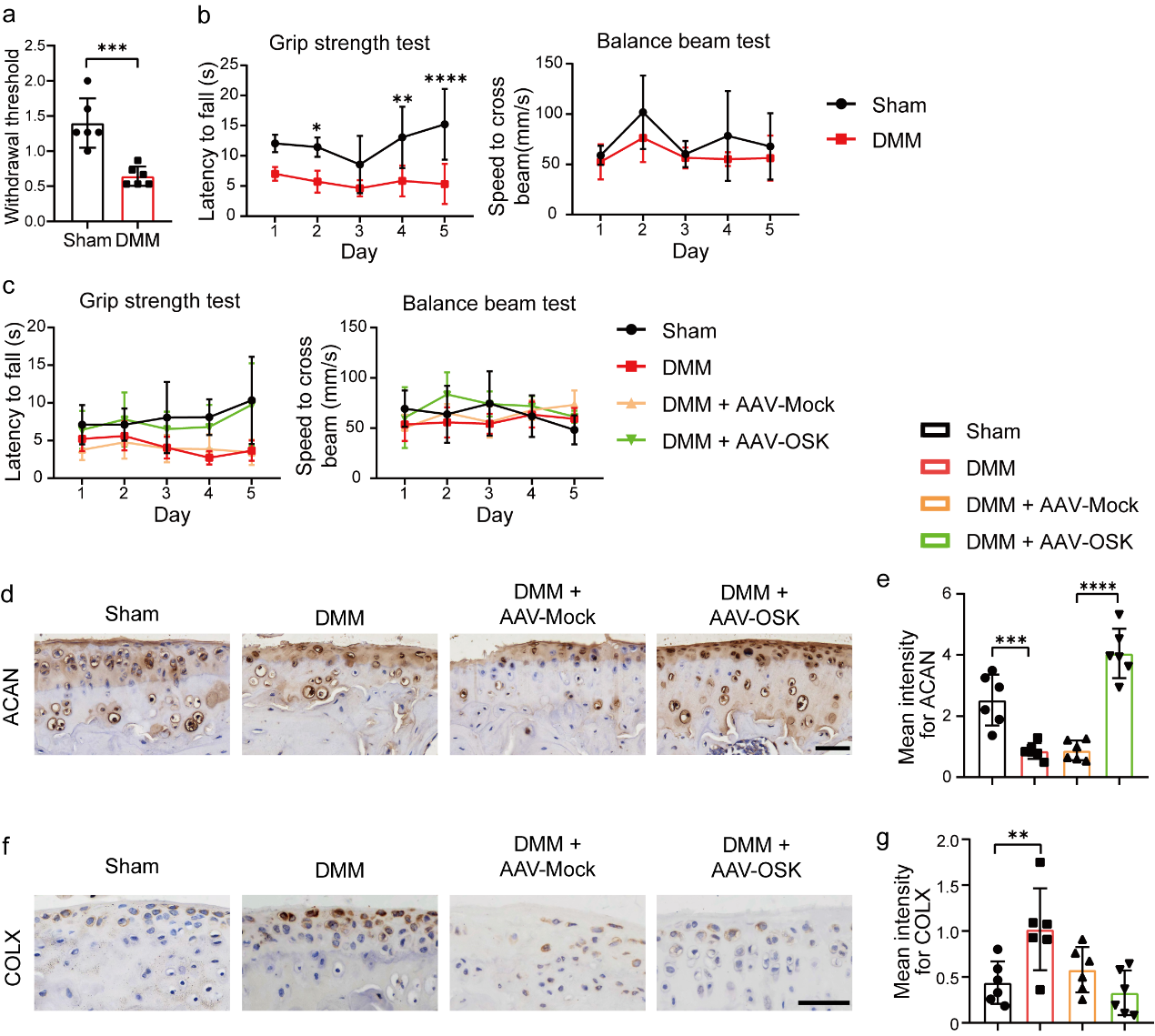


**Supplementary Fig. 12. Behavioral analysis of** **fibrocartilage OA mice model.** (a and b) For Sham and DMM group one month after model induction, the pain was measured by the von Frey assay test (a), the movement was evaluated by the grip strength test and balance beam test for five consecutive days (b). n = 5-6 per group, three repeats for each mouse. Asterisks indicate significant differences between Sham and DMM groups. (c) Movement of mice was evaluated by the grip strength test and balance beam test for five consecutive days. n = 6 per group, three repeats for each mouse. (d-g) Representative immunohistochemical staining of ACAN (d) and COLX (f), and respective quantitative analysis of staining intensity (e and g). Scale bar: 50 μm. n = 6 per group. Data were presented as mean ± SD and analyzed using unpaired student’s t-test (a), two-way ANOVA with Bonferroni test (b and c), or one-way ANOVA with Bonferroni post hoc test (e and g). * *P* < 0.05, ** *P* < 0.01, *** *P* < 0.001, **** *P* < 0.0001.

**Supplementary Table 1. Primer Sequence.**

| **Gene** | **Species** | | **Forward** | | **Reverse** | |
| --- | --- | --- | --- | --- | --- | --- |
| *Gapdh* | mouse | | GCAAGTTCAACGGCACAG | | CGCCAGTAGACTCCACGAC | |
| *Oct4* | mouse | | ACATCGCCAATCAGCTTGG | | AGAACCATACTCGAACCACATCC | |
| *Sox2* | mouse | | ACAGATGCAACCGATGCACC | | TGGAGTTGTACTGCAGGGCG | |
| *Klf4* | mouse | | GTGCCCCGACTAACCGTTG | | GTCGTTGAACTCCTCGGTCT | |
| *c-Myc* | mouse | | ATGCCCCTCAACGTGAACTTC | | CGCAACATAGGATGGAGAGCA | |
| *Nanog* | mouse | | TCTTCCTGGTCCCCACAGTTT | | GCAAGAATAGTTCTCGGGATGAA | |
| *Tnf* | mouse | | CCCTCACACTCAGATCATCTTCT | | GCTACGACGTGGGCTACAG | |
| *Il-1α* | mouse | | CGAAGACTACAGTTCTGCCATT | | GACGTTTCAGAGGTTCTCAGAG | |
| *Il-1β* | mouse | | GAAATGCCACCTTTTGACAGTG | | TGGATGCTCTCATCAGGACAG | |
| *Acan* | mouse | | CCTGCTACTTCATCGACCCC | | AGATGCTGTTGACTCGAACCT | |
| *Col2* | mouse | | CCACACCAAATTCCTGTTCA | | ACTGGTAAGTGGGGCAAGAC | |
| *Mmp13* | mouse | | CTTCTTCTTGTTGAGCTGGACTC | | CTGTGGAGGTCACTGTAGACT | |
| *Col1* | mouse | | ACATGTTCACGTTTGTGGACC | | TAGGCCATTGTGTATGCAGC | |
| *αSma* | | mouse | | GTCCCAGACATCAGGGAGTAA | | TCGGATACTTCAGCGTCAGGA |
| *Runx2* | mouse | | GACTGTGGTTACCGTCATGGC | | ACTTGGTTTTTCATAACAGCGGA | |
| *Osx* | mouse | | AGCGACCACTTGAGCAAACAT | | GCGGCTGATTGGCTTCTTCT | |
| *Tet1* | mouse | | TCAAGCAATGGACCACTGGG | | TCTCCATGAGCTCCCTGACA | |
| *Tet2* | mouse | | ACTCCTGGTGAACAAAGTCAGA | | CATCCCTGAGAGCTCTTGCC | |
| *Tet3* | mouse | | CCGGATTGAGAAGGTCATCTAC | | AAGATAACAATCACGGCGTTCT | |
| *Stat3* | mouse | | CAATACCATTGACCTGCCGAT | | GAGCGACTCAAACTGCCCT | |

**Supplementary Table 2. OARSI Scoring System.**

| Grade | Osteoarthritic damage |
| --- | --- |
| 0 | Normal |
| 0.5 | Loss of Safranin-O without structural changes |
| 1 | Small fibrillations without loss of cartilage |
| 2 | Vertical clefts down to the layer immediately below the superficial layer and some loss of surface lamina |
| 3 | Vertical clefts/erosion to the calcified cartilage extending to <25% of the articular surface |
| 4 | Vertical clefts/erosion to the calcified cartilage extending to 25-50% of the articular surface |
| 5 | Vertical clefts/erosion to the calcified cartilage extending to 50-75% of the articular surface |
| 6 | Vertical clefts/erosion to the calcified cartilage extending >75% of the articular surface |
